# Maternal iron deficiency perturbs embryonic cardiovascular development in mice

**DOI:** 10.1038/s41467-021-23660-5

**Published:** 2021-06-08

**Authors:** Jacinta I. Kalisch-Smith, Nikita Ved, Dorota Szumska, Jacob Munro, Michael Troup, Shelley E. Harris, Helena Rodriguez-Caro, Aimée Jacquemot, Jack J. Miller, Eleanor M. Stuart, Magda Wolna, Emily Hardman, Fabrice Prin, Eva Lana-Elola, Rifdat Aoidi, Elizabeth M. C. Fisher, Victor L. J. Tybulewicz, Timothy J. Mohun, Samira Lakhal-Littleton, Sarah De Val, Eleni Giannoulatou, Duncan B. Sparrow

**Affiliations:** 1 Department of Physiology, Anatomy and Genetics, BHF Centre of Research Excellence, University of Oxford, Oxford, UK; 2 Victor Chang Cardiac Research Institute, Molecular, Structural and Computational Biology Division, Sydney, NSW Australia; 3 Department of Physics, Clarendon Laboratory, University of Oxford, Oxford, UK; 4 Oxford Centre for Clinical Magnetic Resonance Research, John Radcliffe Hospital, Oxford, UK; 5 Department of Clinical Medicine, Aarhus University, Aarhus, Denmark; 6 Heart Development Laboratory, The Francis Crick Institute, London, UK; 7 Advanced Light Microscopy Facility, The Francis Crick Institute, London, UK; 8 Immune Cell Biology and Down Syndrome Laboratory, The Francis Crick Institute, London, UK; 9 Institute of Neurology, University College London, London, UK; 10 Imperial College London, London, UK; 11 Ludwig Institute for Cancer Research Limited, Nuffield Department of Medicine, University of Oxford, Oxford, UK; 12 St Vincent’s Clinical School, University of New South Wales, Sydney, NSW Australia; 13 Present Address: Walter and Eliza Hall Institute of Medical Research, Melbourne, Australia; 14 Present Address: Institute of Developmental Sciences, University of Southampton, Southampton, UK; 15 Present Address: Ealing Hospital, London, UK; 16 Present Address: Department of Biochemistry, University of Cambridge, Cambridge, UK; 17 Present Address: Faculty of Biology, Medicine and Health, University of Manchester, Manchester, UK

**Keywords:** Disease model, Embryogenesis, Experimental models of disease, Risk factors

## Abstract

Congenital heart disease (CHD) is the most common class of human birth defects, with a prevalence of 0.9% of births. However, two-thirds of cases have an unknown cause, and many of these are thought to be caused by in utero exposure to environmental teratogens. Here we identify a potential teratogen causing CHD in mice: maternal iron deficiency (ID). We show that maternal ID in mice causes severe cardiovascular defects in the offspring. These defects likely arise from increased retinoic acid signalling in ID embryos. The defects can be prevented by iron administration in early pregnancy. It has also been proposed that teratogen exposure may potentiate the effects of genetic predisposition to CHD through gene–environment interaction. Here we show that maternal ID increases the severity of heart and craniofacial defects in a mouse model of Down syndrome. It will be important to understand if the effects of maternal ID seen here in mice may have clinical implications for women.

## Abstract (web summary)

From mouse experiments, the authors link iron deficiency in mothers with cardiovascular defects and increased retinoic acid signalling in their offspring, and giving iron early in pregnancy can prevent most defects.

## Introduction

Congenital heart disease (CHD) is the most common class of birth defects, with a global prevalence of 0.9% of live births^1^. It is a major cause of infant mortality and morbidity, and requires ongoing medical treatment throughout life. CHD results from defects occurring early in the development of the embryo, either because of gene mutation, chromosomal abnormality or the impact of environmental factors. Most research in the last 30 years has focused on genetic causes of CHD. However, despite the association of over 100 genes with CHD, mutations in these genes only explain ~30% of cases^2^. Many of the remaining cases of CHD are caused by in utero exposure to environmental factors^3^. Although the teratogenicity of a wide variety of such factors has been known since the 1940s, very little is known about how these factors perturb embryonic development at the molecular level.

In the past 20 years, several populations of cardiac progenitor cells have been identified, and the molecular events driving heart formation in the mammalian embryo have been described in great detail^4^. This knowledge now provides a scaffold for understanding how environmental factors cause CHD. For example, we and others have described at the molecular level in mice how maternal exposure to hypoxia during gestation^5^ or pharmacological activation of HIF1 signalling^6^ causes the most common subtype of CHD, cardiac outflow tract (OFT) defects. However, these hypoxia studies were done in animal models and do not replicate any particular clinical condition, and it is therefore uncertain how relevant these findings are to human populations.

One way in which embryonic hypoxia might occur clinically is through maternal or embryonic anaemia. This is a major global health problem, affecting 20–40% of women of child-bearing age, a total of more than 500 million individuals^7^. Maternal anaemia in rabbits^8^ and maternal iron deficiency (ID) in rats^9^ can result in embryonic lethality, but the molecular mechanisms are unknown. In humans, at least half of all cases of anaemia result from ID^7^. Furthermore, low iron intake during pregnancy in humans may increase the risk of intrauterine growth restriction^10^ and CHD^11^.

In this study, we use a mouse model to investigate how maternal ID affects embryonic development. We show that maternal ID causes severe cardiovascular defects in her offspring. These defects arise from premature differentiation of a subset of cardiac progenitor cells. This effect is likely caused by increased retinoic acid signalling in ID embryos. This is a distinct mechanism to that we previously showed for hypoxia. The defects can be prevented by maternal iron administration early in pregnancy. In addition, they are greatly reduced in offspring of mothers deficient in both iron and the retinoic acid precursor vitamin A; and largely eliminated by mid-gestation administration of an inhibitor of RA signalling, supporting our hypothesis that retinoic acid signalling is elevated in these embryos. Finally, one puzzling feature of many genetic forms of CHD in humans is the considerable variation in penetrance and severity of defects. It has been hypothesised that such variation may be caused by gene–environment interactions. In support of this hypothesis, we show a strong gene–environment interaction in a mouse model of Down syndrome.

## Results

### Maternal iron deficiency perturbs embryonic development

**Fig. 1:**
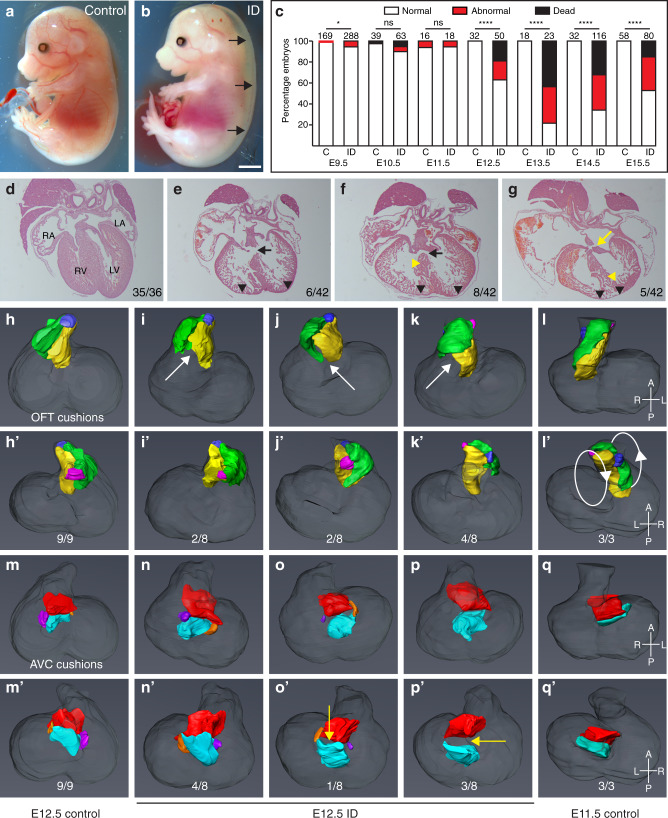
Maternal ID causes embryonic defects and lethality. **a**, **b** Embryos from ID mothers have gross sub-cutaneous oedema. Representative images of control (**a**) and ID (**b**) E15.5 embryos. Black arrows indicate subcutaneous oedema. **c** Histograms of a developmental time-course showing significant embryonic lethality from E12.5. **d**–**g** Representative frontal H&E sections of hearts from control (**d**) and ID (**e**–**g**) E15.5 embryos. VSD (black arrows), AVSD (yellow arrow), thin ventricular myocardium (black arrowheads) and disorganised ventricular septum (yellow arrowheads) are indicated. Relative incidence of each phenotype in all hearts analysed by µMRI, HREM and paraffin sectioning are indicated. **h**–**q** Abnormal cushion formation in E12.5 ID embryos. **h**–**l** Representative 3D Amira reconstructions of OFT cushions from manually segmented HREM data showing dorsal (**h**–**l**) and ventral (**h’**–**l’**) views of (**h**) E12.5 control embryo, (**i**) normally rotated OFT from E12.5 ID embryo, (**j**) partially rotated OFT from E12.5 ID embryo, (**k**) non-rotated OFT from E12.5 ID embryo, (**l**) E11.5 control embryo. The left OFT cushion (yellow), right OFT cushion (green), aortic ICC (pink) and pulmonary ICC (blue) are shown. White arrows indicate unfused proximal cushions. **m**–**q** 3D reconstructions of the AV cushions showing dorsal (**m**–**q**) and ventral (**m’**–**q’**) views from the same embryos. The superior AV cushion (red), inferior AV cushion (cyan), left lateral AV cushion (orange) and right lateral AV cushion (purple) are shown. Yellow arrows indicate non-touching AV cushions. Statistical significance was tested using one-sided Fisher’s exact test (panel **c**). *P* values: E9.5 0.0143; E10.5 0.1735; E11.5 0.7273; E12.5 < 0.0001; E13.5 < 0.0001; E14.5 < 0.0001; E15.5 < 0.0001. ns not significant, *P < 0.05, ****P < 0.0001. Scale bar = 2 mm (**a**, **b**) and 200 µm (**d**–**g**).

We used our previously published model^12^ to investigate the effects of maternal ID on embryonic development. Female C7BL/6 J strain mice were weaned onto, and maintained continuously on, a low iron diet (2–6 ppm). At maturity, these mice had significantly reduced blood haemoglobin (*P* < 0.0001, Supplementary Fig. 1a) and liver iron levels (Fe^56^ concentration *P* = 0.005, Perls staining *P* < 0.0001, Supplementary Fig. 1b–e) compared to females fed a standard diet (200 ppm iron). The range of blood haemoglobin levels observed (20–105 g/l) is comparable to those observed in mild-to-severe anaemia in humans as defined by the World Health Organisation (WHO)^13^. ID females were mated to iron-replete C57BL/6 J males, then maintained on the low iron diet until embryo collection on E15.5. ID embryo morphology was compared to that of embryos from control mothers (Fig. 1a, b). Microscopic Magnetic Resonance Imaging (µMRI) showed that E15.5 embryos had significantly reduced liver iron levels (*P* < 0.0001, Supplementary Fig. 1f–h). Macroscopic observation at dissection revealed that 12/80 ID embryos had died recently (0/58 controls, *P* = 0.0010) and 26/68 of the surviving ID embryos had significant subcutaneous oedema (0/58 controls, *P* < 0.0001).To determine the developmental progression of these phenotypes, we examined ID embryos between E9.5 and E14.5 (Fig. 1c). Oedema and recent death were observed in a significant number of embryos from E12.5 onwards, with a peak at E13.5 (*P* = 0.0008 vs E12.5 and *P* = 0.0078 vs E15.5). We conclude that maternal ID severely perturbs embryonic development in mouse.

### ID embryos have cardiovascular defects

We hypothesised that maternal anaemia causes embryonic hypoxia, which we have previously shown leads to a variety of embryonic defects, most notably in the cardiovascular system^5^. We therefore examined cardiac morphology in surviving ID embryos at E15.5 (Fig. 1d–g, Supplementary Table 1). In total, 30/42 of these embryos had heart defects (1/37 controls, *P* < 0.0001). Membranous and muscular ventricular septal defects (VSDs) were most common (Fig. 1e, f), occasionally coupled with double-outlet right ventricle (DORV) or overriding aorta (OA). Finally, 5/42 embryos had atrioventricular septal defects (AVSD, Fig. 1g, yellow arrow) compared to 0/37 controls (*P* = 0.0377). In addition, the ventricular myocardium was significantly thinner in all ID embryos (Fig. 1e–g, black arrowheads; quantified in Supplementary Fig. 2a, *P* < 0.0001), and in some embryos the ventricular septum (VS) was non-compacted (Fig. 1f, g, yellow arrowheads). The same phenotypes were seen in 7/9 surviving pups analysed at P0, suggesting that it is unlikely that the cardiac defects are simply due to delays in cardiac development.

We next investigated whether there was increased penetrance of abnormal heart phenotype in litters from more severely anaemic dams. The 42 embryos assessed for heart morphology were derived from seven litters. Four dams had haemoglobin levels <80 g/l (equivalent to moderate-severe anaemia in humans) and three dams had ~100 g/l (below the control mean of 132.6 g/l, and equivalent to mild anaemia in pregnant humans). Embryos from dams with <80 g/l had a significantly higher rate of heart defects (19/22) compared to embryos from dams with ~100 g/l (11/20, *P* = 0.0276, one-tailed Fisher’s exact test). Both groups had a significantly increased rate of heart defects to control embryos (*P* < 0.0001, one-tailed Fisher’s exact test).

To investigate the developmental origins of the heart defects, we measured OFT physical parameters at E10.5 using 3D volume-rendered high resolution episcopic microscopy (HREM) datasets (Supplementary Fig. 2b, c). Because OFT morphology changes rapidly, we matched embryos by Theiler stage (TS). In control embryos, distal OFT length increased from TS16 (30–34 somites) to TS18 (40–44 somites), whilst the angle between distal and proximal OFT decreased. By contrast, at TS16 ID embryos had a significantly shorter distal OFT (*P* = 0.0295, Supplementary Fig. 2b), and by TS18, they had a significantly larger distal/proximal OFT angle than controls (*P* = 0.0109, Supplementary Fig. 2c). These observations are similar to mouse models of CHD caused by a reduced contribution of second heart field (SHF) cardiac progenitor cells to the OFT, including *Tbx1*, *Hes1* and *Hoxb1* null embryos, and embryos exposed to hypoxia in utero^5,14–16^. In these studies, reduced OFT length is proposed to cause malrotation and malalignment of the OFT at later developmental stages. In keeping with this hypothesis, by E12.5 4/8 ID embryos had a non-rotated OFT based on cardiac cushion position (Fig. 1k), 2/8 were partially rotated (Fig. 1j) whilst 2/8 were normal (Fig. 1i), compared to 9/9 control embryos with normal OFT rotation (Fig. 1h, *P* = 0.0023) . The non-rotated OFTs in ID embryos resembled those of control E11.5 embryos (Fig. 1l).

We also found both OFT and atrio-ventricular (AV) cardiac cushions were abnormal at E12.5. The cardiac cushions are the precursors of the valves and septa^17^. The OFT cushions form the aortic valve (AoV), pulmonary valve (PV) and the spiral septum dividing the OFT into the aorta and the pulmonary artery^18^. They are also required for the final closure of the tertiary ventricular foramen in the ventricular septum required to functionally separate the left and right ventricles^19^. The AV cushions contribute to the AV valves, as well the atrial and ventricular septa. In the OFT, the two major cushions are elongated and spiral around the OFT. In between the major cushions are two smaller ridges, called intercalating cushions (ICC) or intercalated valve swellings. By E12.5, the major cushions fuse to form the aortico-pulmonary septum as well as the right and left coronary cusps of the AoV and the right and left cusps of the PV. The ICC do not fuse: the aortic ICC forms the non-coronary (NC) leaflet of the AoV, and the pulmonary ICC forms the non-facing (NF) leaflet of the PV. At E12.5, proximal OFT cushion fusion had not occurred in 8/8 ID embryos (Fig. 1i–k white arrows), compared to 2/9 controls (*P* = 0.0019). This was not likely to be due to generalised developmental delay, since ventricular volumes were not significantly different in ID embryos (*P* = 0.0521, Supplementary Fig. 2d), and the appearance of limb morphology and somite number was comparable between E12.5 ID and control embryos. Furthermore, by E13.5, the proximal OFT cushions were still not fused in 5/5 ID embryos (0/5 controls, *P* = 0.0040). Concomitant with the morphological differences in the OFT at E12.5, the right OFT cushion (*P* = 0.0101) and aortic ICC (*P* < 0.001) were significantly smaller in ID embryos (Supplementary Fig. 2f–h), although the left OFT cushion (*P* = 0.3679) and pulmonary ICC (*P* = 0.0650) were not significantly changed (Supplementary Fig. 2e). The aortic ICC was more affected, being only 25% of normal volume, whilst right OFT cushion was 70% normal size.

AV cushion formation was also abnormal in ID embryos. There are four AV cushions. The large inferior and superior cushions contribute to the atrial and ventricular septa, the aortic leaflet of the mitral valve (MV) and the septal leaflet of the tricuspid valve (TV). The smaller right lateral cushion forms the anterior and posterior leaflets of the TV, and the left lateral cushion forms the mural leaflet of the MV. At E12.5, the superior and inferior AV cushion volumes were the same between control and ID embryos (Supplementary Fig. 2i, j). However, the left and right lateral AV cushions were completely absent from 3/8 ID embryos (Fig. 1p; 0/9 controls, *P* = 0.0824), although there was no significant difference in volume between extant lateral cushions in ID embryos and controls (left *P* = 0.4820, right *P* = 0.3636, Supplementary Fig. 2k, l). These cushions were also absent in 3/3 E11.5 control embryos (Fig. 1q), suggesting that this might be due to delayed development. In addition, the inferior and superior cushions were not apposed in 4/8 ID embryos at E12.5, when fusion is normally complete (Fig. 1o, p; yellow arrows; 0/9 controls, *P* = 0.0294). These cushions were still not touching in 3/5 E13.5 ID embryos, suggesting a persistent defect rather than a developmental delay. The presence or absence of AV cushion contact did not correlate well with the extent of OFT rotation, as 3/5 embryos with the most severe OFT rotation defect (Fig. 1k) had normal AV cushion contact, and 2/2 embryos with milder OFT rotation defects (Fig. 1j) had non-touching AV cushions. In normal development, as the aortic root shifts from over the right ventricle to its final position over the left ventricle, the fused proximal OFT cushions align with the growing tip of the muscular VS^18,20^. The remaining interventricular communication is then closed at E14.5 by the membranous VS that forms by fusion of the rightward tips of the AV cushions with the proximal OFT cushions^21^. Delayed or failed proximal cushion fusion, or incomplete shifting of the aortic root, will result in VSD, OA and DORV; and failed OFT-AV cushion fusion will prevent membranous VS formation and can contribute to AVSD^20^. Thus, the cardiac defects observed at E15.5 in ID embryos are likely to arise from the OFT and AV cushion defects observed at E12.5.

**Fig. 2:**
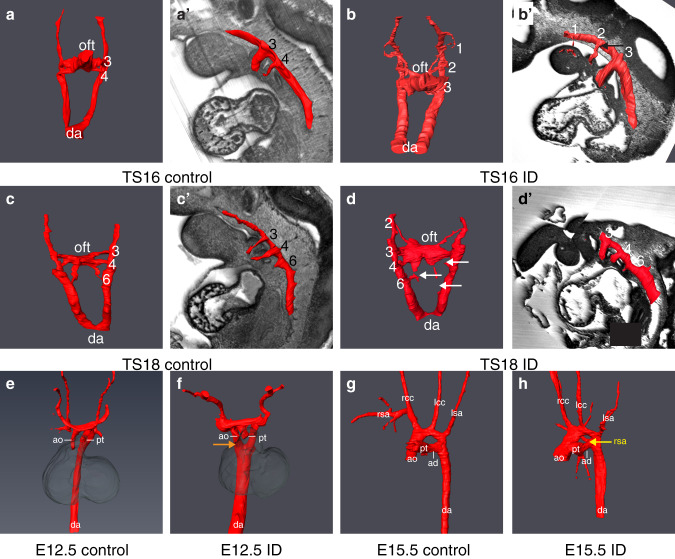
Maternal ID causes aortic arch abnormalities. Comparison of aortic arch artery morphology between control (**a**, **c**, **e**, **g**) and ID (**b**, **d**, **f**, **h**) embryos. Representative 3D Amira reconstructions from manually segmented HREM data of early E10.5 (**a**, **b**), late E10.5 (**c**, **d**), E12.5 (**e**, **f**) and E15.5 (**g**, **h**) embryos. For clarity of PAA identity, 3D models of E10.5 embryos are overlaid onto a sagittal section of the same embryo (**a’**–**d’**). Persistent PAA2 (panel **b’**, black arrow), interrupted PAA4 and PAA6 (panel **d**, white arrows), persistent right dorsal aorta (panel **f**, orange arrow) and aberrant right subclavian artery (panel **h**, yellow arrow) are indicated. da dorsal aorta, oft outflow tract, ao aorta, pt pulmonary trunk, rsa right subclavian artery, rcc right common carotid artery, lcc left common carotid artery, lsa left subclavian artery, ad arterial duct.

Clinically, cardiac OFT defects commonly occur in conjunction with aortic arch (AA) anomalies. Typically, such defects originate from a failure of pharyngeal arch artery (PAA) formation or remodelling from E9.5. During normal development, the first three pairs of PAA form prior to TS16^22^. By TS16, the fourth pair appears, whilst the first two pairs regress, and no longer connect to the dorsal aorta (Fig. 2a). The sixth pair develops by TS18, thus at this stage there are symmetrical arteries in the third, fourth and sixth pharyngeal arches (Fig. 2c). At TS16, whilst all ID embryos had fully patent PAA3, 5/12 ID embryos had bilaterally absent PAA4 (Fig. 2b) and in the remainder, PAA4 was only partially formed on both sides, compared to 7/7 controls with fully patent PAA3-4, (*P* < 0.0001). In addition, in 8/12 embryos the second PAA was still connected to the dorsal aorta (seven bilaterally and one unilaterally; Fig. 2b’, black arrow), compared to 1/7 controls (Fig. 2a, b; *P* = 0.0399). By TS18, whilst all ID embryos had patent PAA3, 2/7 had absent or interrupted PAA4 (one bilateral and one unilateral) and 3/7 had absent or interrupted PAA6 (two bilateral and one unilateral; Fig. 2d, white arrows; 6/6 controls with fully patent PAA3-6; *P* = 0.1224). In addition, 4/7 ID embryos had bilaterally hyperplastic PAA2, with one of these still patent with the dorsal aorta (0/6 controls, *P* = 0.0490). This suggests that PAA development was delayed and abnormal. Indeed, at E12.5, 8/8 ID embryos had persistent right dorsal aorta (Fig. 2f, orange arrow, 0/9 controls, *P* < 0.0001). Unexpectedly, by E15.5 only 5/40 ID embryos had AA anomalies (Supplementary Table 2, 1/37 controls, *P* = 0.1189). These included interrupted AA (IAA), aberrant right subclavian artery (A-RSA, Fig. 2h, yellow arrow), right-sided AA and retroesophageal left subclavian artery (R-LCC, associated with right-sided AA). A similar decrease in the penetrance of AA anomalies between E10.5 and foetal stages has been observed in mouse knockout models^23,24^. This might result from increased lethality of embryos with abnormal AA formation prior to E15.5, or there may be compensatory mechanisms allowing phenotypic recovery. The phenotypes observed at E15.5 are typical of a failure of PAA4 formation^25^. Therefore, the aortic arch defects in ID embryos are likely to arise from the observed perturbation of PAA4 formation earlier in development.

### Maternal ID causes premature differentiation of SHF cardiac progenitor cells

We next investigated the developmental origin of the cardiovascular defects. Four different cell lineages contribute to OFT cardiac cushion formation: endocardium, second heart field (SHF), cardiac neural crest (CNC) and epicardium^17^. Each particular cushion contains a characteristic combination of cells derived from a subset of these lineages. The aortic ICC was most severely effected in ID embryos (Supplementary Fig. 2g). This cushion is predominantly composed of SHF-derived cells^26–28^, suggesting that the reduction in cushion size might be due to a lack of this cell type. A variety of mouse knockout models with cardiac OFT defects have a deficit of anterior SHF cells. This can arise by a variety of processes, including premature differentiation^29^, disruption of proliferation^5,30,31^ or inhibited migration^32^.

**Fig. 3:**
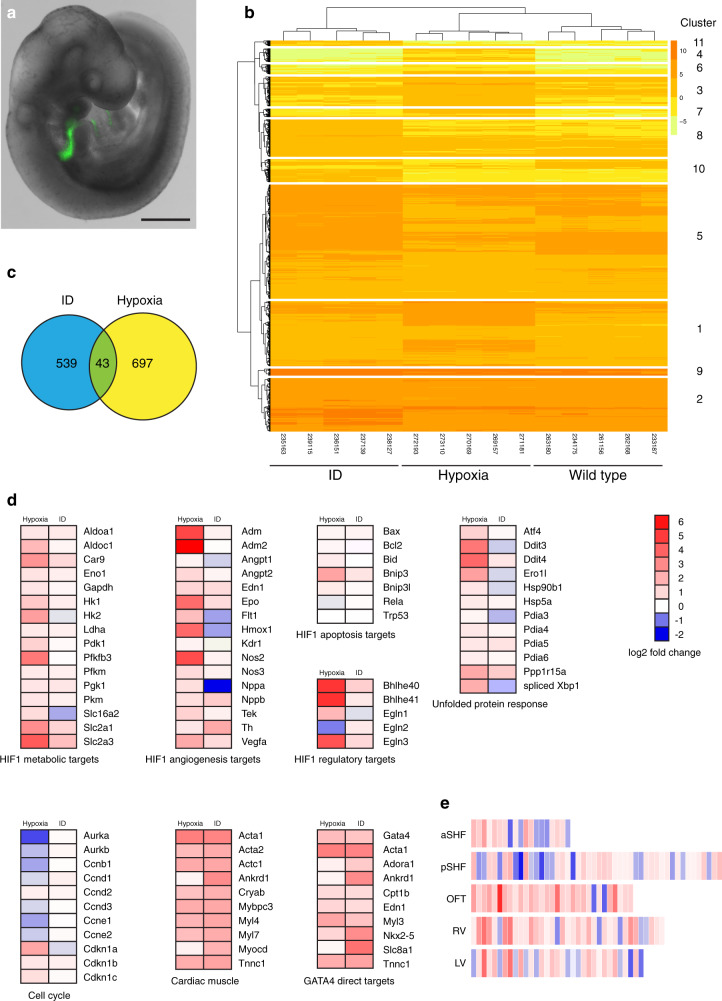
Transcriptomic analysis of anterior SHF cells. **a** Representative E9.5 embryo showing the expression domain of the Mef2c-AHF-GFP transgene (*n* = 5). Scale bar is 500 µm. **b** Unsupervised hierarchical clustering using normalised gene expression levels ordered samples into the correct condition groups. Only differentially expressed genes are shown with adjusted *p* value < 0.01, *B* > 1 and differentially expressed < >2-fold. **c** Comparison of genes differentially expressed relative to control samples shows little overlap between ID and hypoxia samples. Analysis was restricted to genes with adjusted *p* value < 0.01, *B* > 1 and differentially expressed < >2-fold. **d** Comparison of expression changes relative to control samples of ID and hypoxia samples. Heat maps show the average fold-change between ID or hypoxia and control samples of selected genes involved in the hypoxia response, the unfolded protein response, cell cycle control, cardiac muscle proteins and GATA4 direct targets. **e** The ID transcriptome is more closely related to the OFT cluster than the aSHF cluster from single-cell RNA-Seq of heart and cardiac progenitor cells at E9.25 (de Soysa et al.^36^). Heat maps show every gene in each de Soysa cluster with significant (*P* < 0.05) fold-change between ID and control samples. Statistical significance was tested using a hypergeometric test (assuming 20,000 genes in the transcriptome) with Bonferroni correction to control the familywise error rate. *P* values: OFT 3.5798 × 10^−6^; RV 3.2923 × 10^−4^; aSHF 3.5365 × 10^−4^; pSHF 1.8708 × 10^−3^; LV 1.0542 × 10^−2^. aSHF anterior second heart field, pSHF posterior second heart field, OFT outflow tract, RV right ventricle, LV left ventricle.

To investigate if any of these mechanisms were also occurring in ID embryos, we compared the transcriptomes of aSHF cells from ID and control embryos. We also compared these data with the transcriptome of aSHF cells from embryos exposed to hypoxia in utero, the molecular effects of which we have previously described^5^. ID and control female C57BL/6 J mice were mated with hemizygous *Mef2c-AHF-GFP* males^33^. This allele directly drives GFP expression from the *Mef2c*-AHF enhancer element, specifically marking cells strongly in the aSHF and weakly in the distal OFT (Fig. 3a). For control and ID samples, embryos were collected at E9.5. For hypoxia samples, pregnant mice were exposed to an atmosphere containing 6% oxygen for 4 hours on E9.5, and embryos collected immediately after exposure. RNA was isolated from GFP+ cells from five individual somite-matched embryos for each condition and RNA sequencing (RNA-Seq) was performed. Unsupervised hierarchical clustering correctly grouped samples (Fig. 3b). As expected from our previous study^5^, differential expression (DE) analysis showed that aSHF cells from hypoxic embryos had increased expression of unfolded protein response (UPR) genes and spliced *Xbp1* transcript; elevated transcript levels of hypoxia-response genes (including those involved in metabolism, angiogenesis and pathway regulation); increased expression of cell cycle inhibitors; decreased expression of cell-cycle progression genes; but no induction of apoptotic HIF1 targets (Fig. 3d). By contrast, aSHF cells from ID embryos did not activate UPR response genes or HIF1 targets, nor was cell-cycle gene expression altered. Overall, there was very little overlap in the significantly DE genes between ID and hypoxia samples (Fig. 3c). Together, these observations suggest that iron deficiency and hypoxia act via distinct mechanisms to cause heart defects.

We used gene ontology (GO) analysis with the GSEA online resource^34,35^ to provide an unbiased assessment of significantly altered pathways. As expected, the top upregulated gene set in the hypoxia samples was Hallmark_hypoxia (*P* = 5.33 × 10^−55^), and the top downregulated gene set was Hallmark_mitotic_spindle (*P* = 7.66 × 10^−10^). By contrast, the most enriched gene set in the ID model was Hallmark_myogenesis (*P* = 4.65 × 10^−17^). Further analysis of the ID dataset revealed that expression of multiple markers of cardiac OFT differentiation were substantially upregulated (Fig. 3d). We also compared the top significantly DE transcripts from ID embryos with data from single cell transcriptomic analysis of the cardiogenic regions of E9.25 embryos^36^. The ID transcriptome was most closely related to the OFT cluster, rather than to the aSHF cluster (*P* = 3.5798 × 10^−6^, Fig. 3e).

**Fig. 4:**
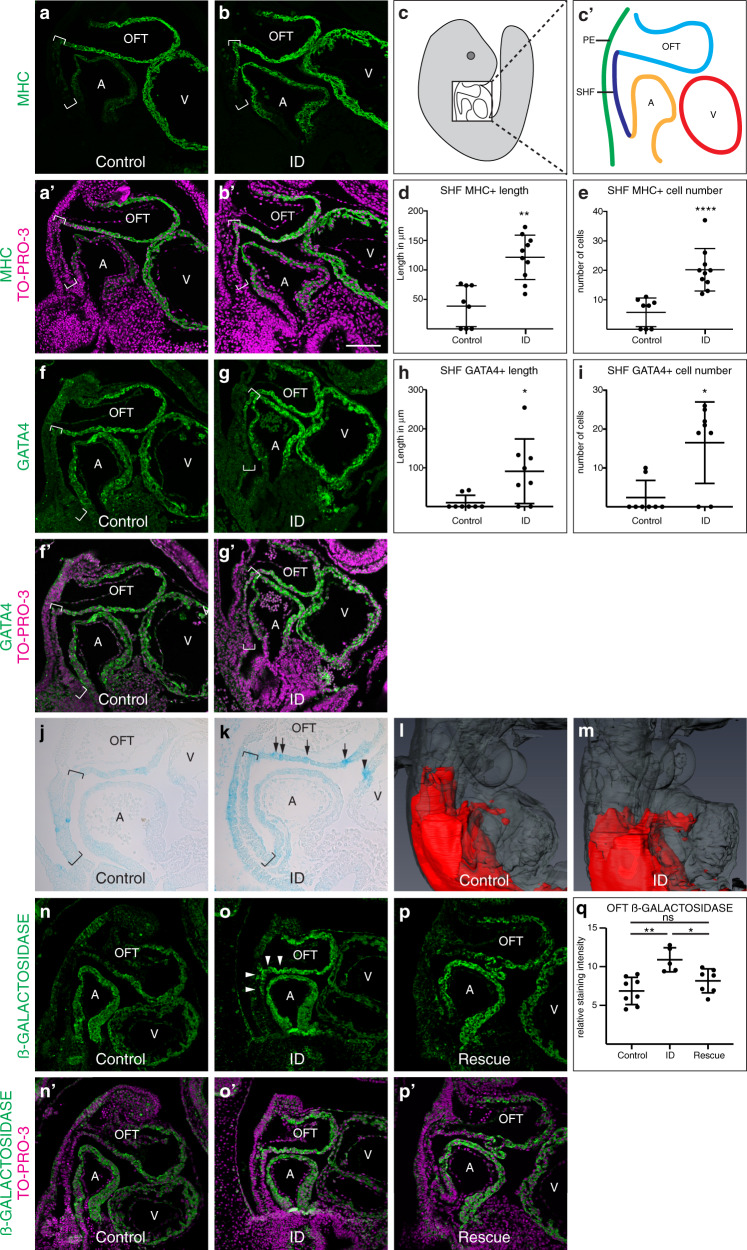
Effects of ID on cardiac progenitors and RA signalling. **a**–**e** Comparison of expression levels of the myocyte differentiation marker MHC (green) in sagittal sections of control (**a**, **a’**) and ID (**b**, **b’**) E9.5 mouse embryos by immunohistochemistry. Nuclei were stained with TO-PRO-3 (magenta). Location of the SHF is indicated by brackets. (**c**, **c’**) Diagrams indicating the relative positions of the SHF (dark blue), pharyngeal endoderm (green), OFT (light blue) and left ventricle (V, red) and left atrium (A, orange) in a sagittal section of an E9.5 embryo. Quantification of length of MHC-positive SHF (**d**) and number of MHC-positive cells in the SHF (**e**) from control (*n* = 8) and ID (*n* = 10) embryos. **f**–**h** Comparison of expression patterns of GATA4 (green) in control (**f**, **f’**) and ID (**g**, **g’**) E9.5 mouse embryos by immunohistochemistry. Nuclei were stained with TO-PRO-3 (magenta). Location of the SHF is indicated by brackets. Quantification of length of GATA4-positive SHF (**h**) and number of GATA-4-positive cells in the SHF (**i**) from control (*n* = 8) and ID (*n* = 8) embryos. **j**–**m** Comparison of RA signalling levels in control (**j**, **l**) and ID (**k**, **m**) E9.5 embryos carrying the RARE-LacZ reporter allele. **j**, **k** Sagittal sections of E9.5 embryos stained with X-gal in wholemount. Location of the SHF is indicated by brackets. Increased and patchy staining in the OFT (black arrows) and increased staining in the ventricle (black arrowheads) are indicated. **l**,**m** Representative 3D Amira reconstructions of X-gal staining (red) from automatically thresholded two-channel HREM data derived from E9.5 embryos stained in wholemount. The embryos are shown in transparent grey. **n**–**q** Comparison of ß-GALACTOSIDASE protein expression (green) by immunohistochemistry in control (**n**, **n’**), ID (**o**, **o’**) and E7.5 rescue (**p**, **p’**) E9.5 embryos carrying the RARE-LacZ reporter allele. Nuclei were stained with TO-PRO-3 (magenta). Increased staining in the OFT and SHF (white arrowheads) and is indicated (**q**) Quantitation of the relative ß-GALACTOSIDASE staining intensity in the OFT of control (*n* = 8), ID (*n* = 5) and E7.5-rescue (*n* = 7) embryos. Graphs show mean ± standard deviation. Statistical significance was tested by two-sided Mann–Whitney *U* test (panels **d**, **e**, **h**, **i**) or one way ANOVA with Tukey’s post hoc test adjusted for multiple comparisons (panel **q**). *P* values: 0.0013 (panel **d**); <0.0001 (panel **e**); 0.0120 (panel **h**); 0.0120 (panel **i**); 0.0014, 0.3058, 0.0298 (panel **q**). ns not significant, *P < 0.05, **P < 0.01, ****P < 0.0001. Scale bar = 130 µm (**a**, **b**, **f**, **g**, **n**, **o**, **p**) and 95 µm (**j**, **k**).

Premature differentiation of the aSHF is a feature of several mouse knockout models with similar cardiac defects to our ID model, including *Tbx1* null^29,37^ and *Hoxb1* null^16^ embryos. In these models, premature differentiation of aSHF cells at E9.5 causes similar phenotypes to ID, namely defective OFT elongation, alignment and septation, resulting in a specific set of cardiac defects by E15.5, including membranous VSDs (with or without DORV or OA), transposition of the great arteries (TGA) and persistent *truncus arteriosus* (PTA), as well as AA anomalies. We validated the presence of premature differentiation in the SHF by examining protein expression levels and pattern of the myocyte differentiation marker MHC (Fig. 4a–e). MHC expression is normally restricted to the wall of the OFT, with no expression in the contiguous cells of the aSHF (Fig. 4a). By contrast, in ID embryos ectopic MHC expression extended into the aSHF (Fig. 4b, d), and a significantly larger number of aSHF cells expressed MHC (*P* < 0.0001, Fig. 4e). Similar defects can be caused by reduced cell proliferation^38,39^ in the SHF. However, there was no significant difference in the percentage of phosphorylated histone H3-positive nuclei between ID and control SHF cells (*P* = 0.3438, Supplementary Fig. 3a, b, d).

The cardiac transcription factor GATA4 is a key regulator that promotes the switch from proliferation to differentiation in SHF cells^29^. *Gata4* transcripts were substantially increased in the ID aSHF transcriptome (Fig. 3d), and GATA4 protein and transcript levels were elevated in the OFT and ectopically expressed in the aSHF of ID embryos at E9.5 (Fig. 4f–I, Supplementary Fig. 3e, f). By contrast, the expression levels and domain of TBX5 protein, a marker of the posterior SHF, were unchanged (Supplementary Fig. 3g–i). GATA4 directly activates the transcription of many cardiac differentiation genes and transcription factors, including MHC^40^. Many of these direct GATA4 targets were also substantially upregulated in the ID aSHF transcriptome (Fig. 3d). In *Tbx1* null embryos, *Gata4* expression is elevated, and SHF cells prematurely differentiate^29^. Thus, it is likely that the cardiovascular defects in the ID embryos result from premature differentiation of aSHF cells, which then fail to migrate into the OFT, OFT cushions and aortic arches.

### Embryos show perturbed retinoic acid signalling

The cardiovascular defects observed in ID embryos resemble those present in mouse or chick embryos with excess retinoic acid (RA) signalling^41–43^, after exposure to excess vitamin A^44^, or in humans exposed to the drug isotretinoin^45^. In mouse embryos, RA signalling is mediated by all-trans retinoic acid (ATRA). This compound is synthesised from vitamin A, and can be subsequently catabolised to the biologically inactive compound 4-hydroxy ATRA by the CYP26 family of cytochrome P450 enzymes. The active site of CYP26 enzymes incorporates haem, so we hypothesised that RA catabolism might be reduced in ID embryos, resulting in excess RA signalling. Supporting this hypothesis, E15.5 *Cyp26b1* null mouse embryos have a similar spectrum of heart defects to ID embryos, including membranous VSDs (frequently coupled with DORV or OA), partial AVSDs and AA anomalies (C. Roberts, personal communication). In addition, *Gata4* transcription is directly activated by RA signalling^46,47^, thus this might also explain the ectopic upregulation of *Gata4* in ID embryos.

To test this hypothesis, we examined the level of RA signalling in ID embryos using the RARE-LacZ transgenic reporter^48^. Males carrying the RARE-LacZ allele were crossed with control or ID C57BL/6 J females, embryos collected at E9.5, and stained for ß-GALACTOSIDASE activity with X-gal. In control embryos at E9.5, ß-GALACTOSIDASE activity was present in paraxial mesoderm, SHF, OFT and brain, as previously described^48^. ID embryos had a broadly similar expression pattern, however X-gal staining developed more rapidly, suggesting higher levels of RA signalling in ID embryos. Sections from somite-matched embryos dissected on the same day and stained for the same time confirmed that ID embryos had stronger OFT expression than controls (Fig. 4j, k). In addition, expression was patchy in ID embryos (Fig. 4k, arrows) and was also present in the ventricle (Fig. 4k, arrowhead). Two-channel HREM of X-gal-stained E9.5 embryos coupled with 3D reconstruction in Amira was used better visualise the 3D pattern of RA expression in the OFT (Fig. 4l, m). This shows that RA signalling levels were present in a larger domain of the OFT. We confirmed the changes in ß-GALACTOSIDASE protein expression in the OFT using immunofluorescence (Fig. 4n, o, q). Finally, to confirm that RA signalling directly regulates *Gata4* transcription, we examined published ChIP-seq data and identified a putative RA-responsive enhancer in the first intron of the mouse *Gata4* gene (Supplementary Fig. 4). In cardiomyocytes derived from differentiated mouse ESC, H3K27ac marks a region that is flanked by two consensus RAR/RXR binding sites^49^. These sites bind RARA and RXRA in in response to RA-induced differentiation of F9 embryo carcinoma cells^50^.

These data suggest that ID embryos have increased RA signalling in the SHF and OFT, directly causing ectopic activation of *Gata4* in the SHF and initiating premature differentiation of these cells.

### Other embryonic processes dependent on RA signalling are perturbed in ID embryos

In addition to cardiovascular development, RA signalling has many other roles throughout embryonic development^51^. To determine if ID causes a more general upregulation of RA signalling in the developing embryo, we investigated if the development of any other RA-dependent systems were also perturbed. We first investigated the origins of the subcutaneous oedema observed at E15.5. This is a relatively common phenotype in embryonic lethal and sub-viable knockout mouse models. For example, in an unbiased survey of embryonic lethal mouse strains, 24/42 had subcutaneous oedema at E14.5^52^. In humans, this phenotype is called *hydrops fetalis* and can arise from embryonic anaemia or cardiovascular defects, resulting in cardiac failure and a generalised fluid build-up^53^. In our study, we assessed 42 ID E15.5 embryos for both oedema and heart defects. 26 were concordant and 16 discordant, thus there was no correlation between the presence of oedema and heart defects (*P* < 0.0001).

**Fig. 5:**
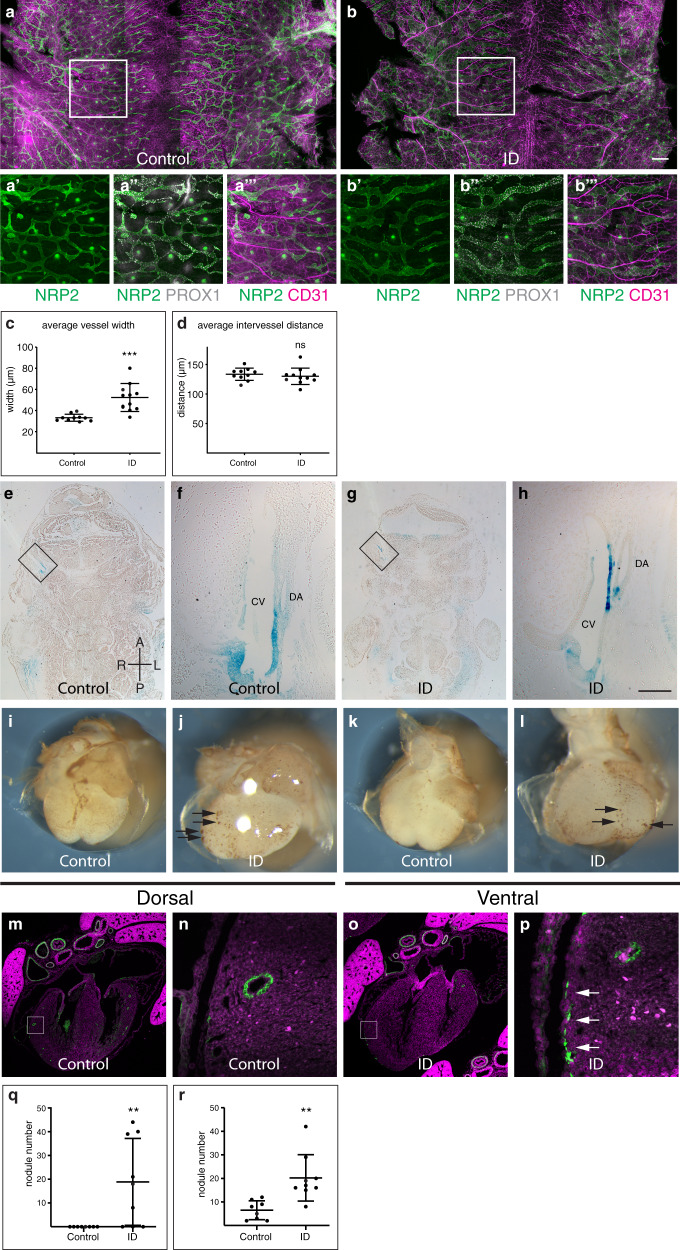
Lymphatic and coronary vasculature development is perturbed in ID embryos. **a**–**m** Lymphatic development. **a**–**d** Comparison of NRP2, PROX1 and CD31 expression in back skin from control (**a**) and ID (**b**) E14.5 embryos. Magnified views of the boxed areas are shown in panels **a’**–**a”’** and **b’**–**b”’**, respectively (see also Supplementary Fig. 5). Quantification of the average lymphatic vessel width (**c**) and average intervessel distance (**d**) in control (*n* = 10) and ID (*n* = 11) embryos. **e**–**h** Comparison of RA signalling levels in frontal sections of control (**e**) and ID (**g**) E11.5 embryos carrying the RARE-LacZ transgene. (**f**, **h**) Magnified views of the boxed areas in (**e**) and (**h**), respectively. **i**–**r** Coronary vasculature development. Comparison of CD31 staining of E14.5 hearts from control (**i**, **k**) and ID (**j**, **l**). Black arrows indicate endothelial nodules. Quantitation of endothelial nodule number on the dorsal (**q**) and ventral (**r**) surfaces of control (*n* = 8) and ID (*n* = 9) hearts. **m**–**p** Comparison of CD31 (green) expression in the coronary vasculature of control (**m**) and ID (**o**) E17.5 embryos. Nuclei were stained with TO-PRO-3 (magenta). Magnified views of the boxed areas are shown in panels (**n**) and (**p**), respectively. White arrows indicate ectopic CD31 staining. Graphs show mean ± standard deviation. Statistical significance was tested by two-sided unpaired *t*-test with Welch’s correction (panels **c**, **r**), two-sided unpaired *t*-test (panel **d**) or two-sided Mann–Whitney *U* test (panel **q**). *P* values: 0.0006 (panel **c**); 0.5299 (panel **d**); 0.0090 (panel **q**); 0.0028 (panel **r**). ns not significant, **P < 0.01, ***P < 0.001. Scale bar = 715 μm (**a**, **b**); 370 µm (**a’**–**b”’**); 755 µm (**e**, **g**); 95 µm (**f**, **h**); 680 µm (**i–l**); 640 µm (**m**, **o**); 64 µm (**n**, **p**). CV cardinal vein, DA dorsal aorta.

Alternatively, oedema can be caused by a failure of normal lymphatic development. This process requires RA signalling, and genetic knockout of *Cyp26b1* results in an identical phenotype to that observed in ID embryos^42^. Therefore, we investigated whether lymphatic development was compromised in ID embryos. The blood and lymphatic vasculature were visualised in E14.5 dorsal back skin using CD31, PROX1 and NRP2 antibodies^54^ (Fig. 5a, b). Lymphatic vessels in ID embryos had significantly increased in vessel diameter (*P* = 0.0006, Fig. 5c) and the vessels had more PROX1-positive nuclei (compare Fig. 5 panels a” and b”). The degree of increase in lymphatic vessel diameter (33 to >52 μm) was similar to other mouse models with perturbed lymphatic development^42,55^. By contrast, inter-vessel distance was not significantly altered (*P* = 0.5299, Fig. 5d), nor were there changes in the patterning of the blood vasculature (Fig. 5a”’, b”’).

These data suggest that the changes in lymphatic vasculature may be due to a primary defect in lymphatic development, rather than to increased dilation of the vessels subsequent to cardiac failure. In control E11.5 embryos, RA signalling is activated between the dorsal aorta and the ventro-medial side of the cardinal vein (Bowles et al.;^22^ Fig. 5e, f). This is on the opposite wall of the cardinal vein from where lymphatic endothelial cell (LEC) progenitors arise. In ID embryos, RA signalling was increased and expanded dorsally (Fig. 5g, h). This is indistinguishable from the phenotypes observed in *Cyp26b1* null embryos, where RA signalling is also increased^42^.

We next examined the development of the coronary vasculature. This develops through a stepwise vasculogenic programme. Firstly, an immature vessel plexus forms, and then this is remodelled into a mature vascular bed^56^. The coronary endothelial progenitors arise mostly from the *sinus venosus* (SV) and the endocardium^57^. The epicardium, a layer of cells that migrates over the heart surface from E9.0, is also required for coronary vascular development^58^. It secretes trophic factors, including RA, that stimulate myocardial growth and coronary plexus development. It also contains epicardial-derived progenitor cells (EPDCs) that give rise to cardiac fibroblasts and vascular smooth muscle cells (VSMC) that stabilise the coronary vasculature. EPDCs also contribute to the lateral AV cushions^59^, which were missing in some ID embryos (Fig. 1p). In total, 8/8 control hearts at E14.5 had normal coronary plexus formation on both dorsal and ventral surfaces (Fig. 5i, k). By contrast, 8/8 ID hearts showed a reduced plexus area, and large numbers of endothelial nodules were present on both heart surfaces (Fig. 5j, l, black arrows). This is similar to models of perturbed coronary vascular development due to increased RA signalling^60,61^.

By E17.5, 6/6 control E17.5 hearts had clear CD31-positive endothelial tubes throughout the myocardium (Fig. 5m, n). By contrast, 6/6 E17.5 ID embryos had fewer obvious endothelial tubes in the myocardium. Instead, patches of CD31-positive cells were visible on the surface of the heart (Fig. 5p, white arrows). This phenotype is very similar to that of *Dhrs3* null embryos with increased RA signalling^61^. However, despite the abnormal patterning of distal coronary vessels, the patterning of the proximal coronary vessels was normal. In total, 15/16 E15.5 ID embryos had correctly positioned coronary ostia, although 6/16 had a single additional coronary ostia unilaterally (one right and five left), in line with the previously noted incidence in the C57BL/6 J strain^62^.

In the absence of normal epicardial formation, myocardial growth is often reduced. Fittingly, ventricular compact myocardium thickness was significantly reduced in E15.5 ID embryos (*P* < 0.0001, Supplementary Fig. 2a). Typically, embryos with faulty epicardium and/or coronary vascular development die in utero between E12.5–15.5, thus these phenotypes might further contribute to the embryonic lethality in ID embryos in association with the aortic arch abnormalities described above.

Finally, somite segmentation is also regulated by RA signalling. Excess RA causes abnormalities in vertebral patterning and delayed ossification^41^. We therefore examined the developing skeletal cartilage in E14.5 embryos by alcian blue staining. In total, 29/33 ID embryos had mild vertebral segmentation defects, including fused lamina, missing pedicles and split vertebral bodies (Supplementary Fig. 5d, 0/21 controls, *p* < 0.0001).

In summary, ID embryos have defects in a variety of tissues that require RA signalling for normal patterning, and these defects are similar to those in genetic models of increased RA signalling. Thus, our data supports the hypothesis that ID results in a wide-spread disruption of RA signalling in the developing embryo.

### Dietary supplementation mid-gestation rescues the heart and lymphatic phenotypes

**Fig. 6:**
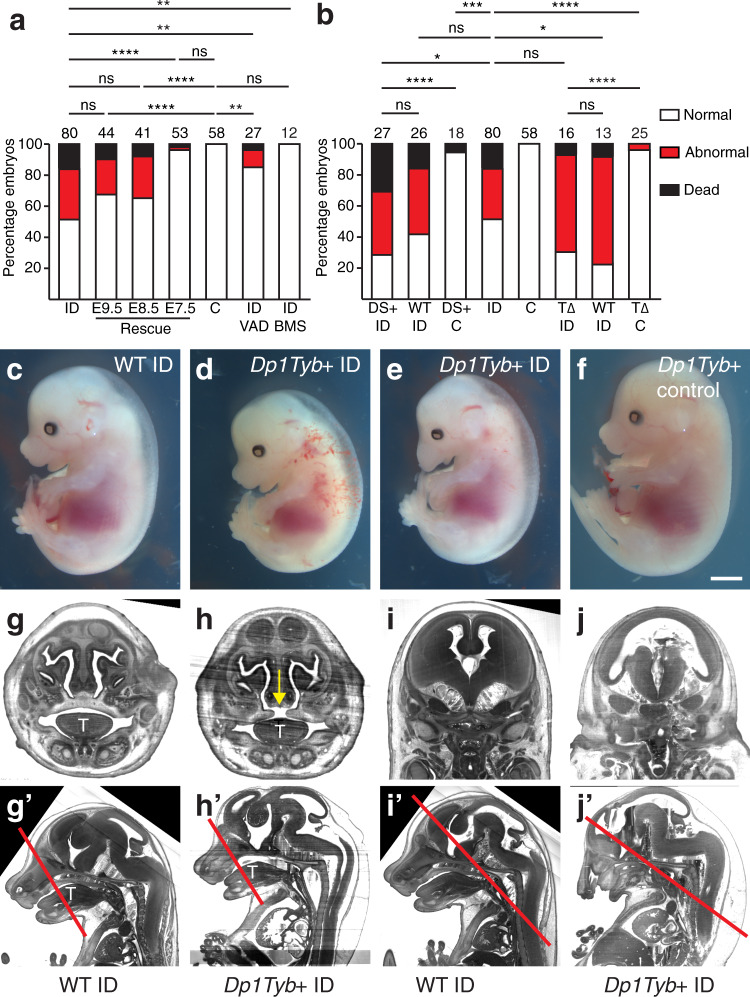
Phenotypic rescue and gene-environment interaction. **a** Phenotypic rescue. Pregnant mice were returned to iron-replete diet between E7.5-E9.5; fed continuously on an iron and vitamin A deficient diet (ID/VAD); or fed continuously on an iron deficient diet and treated with 0.7 mg/ml BMS493 on E8.5 and E9.5 (ID/BMS). Histograms showing the percentage of embryos that were dead, abnormal or normal at E15.5. **b** Investigation of gene–environment interaction. Iron deficient (ID) and control (C) C57BL/6 J females were crossed with males carrying the *Tbx1*^*null*^ allele (T∆), or the *Dp1Tyb* duplication (DS+). Histograms showing the percentage of embryos that were dead, abnormal or normal at E15.5. **c**–**f** Representative images of *Dp1Tyb*+ control (**c**), wild type control ID (**d**), *Dp1Tyb*+ ID with holoprosencephaly (**e**), *Dp1Tyb*+ control (**f**), E15.5 embryos. **g**–**j** *Dp1Tyb*+ embryos from ID mothers have craniofacial defects. Representative 3D reconstructed frontal sections of HREM data from wild type ID (**g**, **i**) and *Dp1Tyb* + ID (**h**, **j**) E15.5 embryos showing examples of failed secondary palate fusion (**h**, yellow arrow) and holoprosencephaly (**j**). **g’**–**j’** Sagittal sections from the same embryos showing the location of the sections in (**g**–**j**) (red lines). note that panels (**g**) and (**i**) are the same control embryo. T tongue. Statistical significance was tested using one-sided Fisher’s exact test (panel **c**). *P* values: 0.0657 (ID vs E9.5); 0.1127 (ID vs E8.5); <0.0001 (ID vs E7.5); 0.0019 (ID vs ID VAD); 0.0009 (ID vs ID BMS); < 0.0001 (C vs E9.5); <0.0001 (C vs E8.5); 0.2257 (C vs E7.5); 0.0087 (C vs ID VAD); >0.9999 (C vs ID BMS); 0.4859 (DS + ID vs WT ID); <0.0001 (DS + ID vs DS + C); 0.0321 (DS + ID vs ID); 0.2494 (WT ID vs ID); (0.0006 DS + C vs ID); 0.4742 (T∆ ID vs WT ID); <0.0001 (T∆ ID vs T∆ C); 0.1000 (T∆ ID vs ID); 0.0457 (WT ID vs ID); <0.0001 (T∆ C vs ID). ns not significant, *P < 0.05, **P < 0.01, ***P < 0.001, ****P < 0.0001.Scale bar = 2 mm (**c**–**f**); 700 µm (**g**, **h**); 1.2 mm (**I**, **j**); 1.6 mm (**g’**–**I’**).

Clinically, ID in pregnant women can be treated rapidly and effectively in many cases^63^. Therefore, it would be useful to know if, and when, during pregnancy that iron supplementation might rescue embryonic defects. We transferred pregnant ID mice from low iron to normal diet (200 ppm iron) 7–9 days post mating. In each case, maternal haemoglobin levels returned to normal by E15.5 (Supplementary Fig. 6a). Diet change on days 8 (*P* = 0.1241) or 9 (*P* = 0.0735) had no significant effect on the prevalence of either oedema or embryonic lethality at E15.5 (Fig. 6a). By contrast, diet change on day 7 resulted in a significant reduction in both oedema and embryonic lethality (2/53 embryos abnormal or dead, compared to 38/80 ID embryos, *P* < 0.0001; and 0/58 controls, *P* = 0.2257; Fig. 6a, Supplementary Fig. 6d). Furthermore, 0/26 of viable E7.5-rescued embryos examined at E15.5 had heart defects (30/42 ID embryos, *P* < 0.0001; and 1/37 controls, *P* = 0.5873; Supplementary Table 1), indicating a complete rescue of the heart phenotype in these embryos. Analysis of E7.5-rescued embryos carrying the RARE-LacZ transgene confirmed that RA signalling levels returned to normal in the OFT of these embryos at E9.5 (Fig. 4p, q). Iron uptake from the gut is swift, taking only a few hours^64^, and is even faster in ID animals with low hepcidin levels^65^. Once in the maternal bloodstream, iron is transferred to the embryo in <6 h^66^. Thus, phenotypic rescue by returning mothers to the iron-replete diet on E7.5, but not later, suggests that the critical period of embryonic development that is sensitive to ID is approximately E8.5. This is identical to studies of RA exposure, where embryonic heart development is most vulnerable at E8.5^67–69^. It is also broadly similar to our previous studies of embryonic hypoxia, which showed a peak in vulnerability of SHF cells to hypoxia at E9.5^5^. This stage of development is when SHF cells migrate into the OFT and when pharyngeal arch morphogenesis takes place. It also corresponds to the initial movement of epicardial cells to the heart’s surface^70^ and the induction of lymphatic endothelial cells in the wall of the cardinal veins^71^. Thus, restoration of normal RA signalling at this stage is likely to explain the rescued cardiac, lymphatic and coronary vessel defects.

### Dietary or pharmacological reduction of RA signalling rescues the heart and lymphatic phenotypes

We next tested our mechanistic hypothesis by further altering the maternal diet. RA is produced from dietary vitamin A. It has been known since the 1930s that maternal vitamin A deficiency (VAD) disrupts development^72^, and this is now known to be caused by reduced embryonic RA signalling. We reasoned that embryos of a mother fed a diet low in iron (leading to increased RA signalling) and deficient in vitamin A (leading to reduced RA signalling) might result in relatively normal levels of RA, and thus restore normal embryonic development. Female C7BL/6 J strain mice were weaned onto, and maintained continuously on, a diet containing low iron (2–6 ppm) and no added vitamin A (control and ID diets both have 15 IU/g vitamin A). As before, these mice had significantly reduced blood haemoglobin, confirming that VAD did not affect iron metabolism (*P* = 0.0005, Supplementary Fig. 6a). These mice were mated to control C57BL/6 J males, then maintained on the ID/VAD diet until embryo collection on E15.5. µMRI imaging confirmed that embryos had reduced liver iron levels (Supplementary Fig. 6i). Strikingly, only 1/27 embryos were dead and 3/27 had oedema (Fig. 6a; Supplementary Fig. 6e). This is a significantly lower rate of death and abnormality than ID embryos (38/80 ID embryos, *P* = 0.0019), although not a complete phenotypic rescue (0/58 controls, *P* = 0.0087). Furthermore, only 3/25 of these embryos had heart defects, compared to 30/42 ID (*P* < 0.0001) and 1/37 control embryos (*P* = 0.1752), indicating an almost complete rescue of heart defects (Supplementary Table 1).

To provide more evidence in support of our hypothesis, we tested if pharmacological reduction of RA signalling was also able to rescue the defects in ID embryos. We treated pregnant ID C57BL/6 J mice with two 0.7 mg/kg doses of the inverse pan-retinoic acid receptor agonist BMS 493^73^ on E8.5 and E9.5, and harvested embryos at E15.5. 0/12 low dose ID embryos were dead and 0/12 had oedema (Fig. 6a; Supplementary Fig. 6f). This is a significantly lower rate of death and abnormality than untreated ID embryos (38/80 ID embryos, *P* = 0.0009), and suggests a significant phenotypic rescue (0/58 controls, *P* > 0.9999). Furthermore, only 1/12 of the low dose ID embryos had heart defects, compared to 30/42 ID (*P* = 0.0001) and 1/37 control embryos (*P* = 0.4337), indicating this treatment also significantly rescued the heart defects (Supplementary Table 1). Finally, to confirm inhibitor activity, we treated ID pregnant females with two 3.5 mg/kg doses at E8.5 and E9.5. In total, 9/9 of these embryos had severe sub-cutaneous oedema and 8/9 had coloboma (Supplementary Fig. 6g). Furthermore, 7/8 hearts analysed had common arterial trunk (CAT). Coloboma and CAT are typical phenotypes of high dose BMS493 treatment and a reduction of RA signalling^73,74^, but were not observed in either ID embryos or ID embryos treated with a low dose of BMS493. This confirms that BMS493 was active in our experiments.

In conclusion, both dietary and pharmacological reduction of RA signalling in ID embryos rescues the heart and lymphatic defects, providing further evidence that these defects are due to increased RA signalling.

### Gene–environment interactions increase the penetrance and expressivity of embryonic defects

In human CHD, even in families with a known monogenic cause, there is often an array of different cardiac defects between individuals with the same causative mutation (variable expressivity), while others with the same mutation do not develop CHD at all (variable penetrance)^75^. This suggests that CHD phenotypes are commonly affected by genetic or environmental modifiers. Previously we have shown in mouse that short-term gestational hypoxia is one such modifier, increasing the prevalence and severity of heart defects in genetically susceptible embryos, as well as causing heart failure and embryonic lethality^76,77^. We hypothesised that ID might cause a similar gene–environment interaction (GxE). Two of the most common human genetic syndromes that include CHD with incomplete penetrance are 22q11.2 deletion syndrome^78^ and Down syndrome (DS)^79^. In both cases, the types of heart defects presented clinically are similar to those of the ID model. Furthermore, in *Tbx1* null embryos all three *Cyp26* genes are downregulated, suggesting that RA signalling may be altered in 22q11.2 deletion syndrome^43^. It has long been appreciated that there is considerable variation in the severity and penetrance of cardiovascular phenotypes in human patients with either syndrome. To test the hypothesis that this variation might be controlled by an environmental factor such as ID, we crossed male C57BL/6 J background mice heterozygous for either a *Tbx1* null allele^80^ (a model of 22q11.2 deletion syndrome), or the *Dp1Tyb* duplication^81^ (a model of DS), with ID females and analysed embryonic phenotypes at E15.5. In both cases, we confirmed that maternal haemoglobin levels were significantly reduced (*Tbx1* *P* = 0.0277, *Dp1Tyb* *P* = 0.0035, Supplementary Fig. 6a) and that embryos had reduced liver iron levels (Supplementary Fig. 6j, k). There was no increase in the prevalence of embryonic death and oedema, heart defects or aortic arch abnormalities in *Tbx1*^*+/null*^ ID embryos (Fig. 6b; Supplementary Table 1; Supplementary Table 2). Thus, maternal low iron status is not likely to be a risk factor for increasing penetrance or severity of cardiovascular phenotypes in 22q11.2 deletion syndrome.

Similarly, E15.5 ID embryos carrying the *Dp1Tyb* duplication (*Dp1Tyb* + ID) had the same prevalence of death and abnormality as wild type ID control littermates (Fig. 6b, 19/27 *Dp1Tyb* + ID, 15/26 wild type ID littermates, *P* = 0.2498). Strikingly however, affected embryos had more severe subcutaneous oedema, and their lymphatics were more frequently blood-filled (Fig. 6d; 10/11 *Dp1Tyb* + ID oedemic embryos with blood-filled lymphatics compared to 4/11 wild type ID, *P* = 0.0119). In addition, we observed craniofacial defects in surviving *Dp1Tyb* + ID embryos. 10/19 of these embryos had a failure of secondary palate fusion (1/39 wild type ID controls, *P* < 0.0001, Fig. 6h), and a further 2 embryos had holoprosencephaly (Fig. 6e, j). By contrast, only 1/18 *Dp1Tyb* + control embryos was dead at E15.5, and none of the survivors had oedema, holoprosencephaly or a failure of secondary palate fusion (Fig. 6f). This induction of previously undescribed phenotypes is an indication that we have identified a *bona fide* gene–environment interaction, rather than a simple additive effect.

We also assessed heart morphology in surviving embryos at E15.5. 16/18 ID *Dp1Tyb* + ID embryos had heart defects (Supplementary Table 1), compared to 10/19 wild type ID littermates (*P* = 0.0186) and 1/16 *Dp1Tyb* + controls (*P* < 0.0001). Furthermore, *Dp1Tyb* + ID embryos had significantly more AVSDs (10/18) than wild type ID embryos (3/19, *P* = 0.0135), *Dp1Tyb* + control embryos (0/16, *P* = 0.0003) or ID embryos (5/42, *P* = 0.0008). Finally, 4/18 *Dp1Tyb* + ID embryos had type I PTA, which was never observed in wild type ID control, *Dp1Tyb* + control or wild type ID control embryos. Thus, we conclude that maternal ID leads to an increase in the penetrance and severity of heart defects in *Dp1Tyb* + mouse embryos. By contrast, there was no change in the prevalence of AA anomalies between *Dp1Tyb* + ID and wild type ID embryos (Supplementary Table 2).

In conclusion, ID is a substantial modifier of heart, lymphatic and/or craniofacial phenotype in a mouse model of DS.

## Discussion

Here we report a previously unknown environmental teratogen in mice: maternal ID anaemia. Clinically, this is potentially of importance, since anaemia is a major global health problem affecting 38% of pregnant women, half of which is due to ID, the most common micronutrient deficiency worldwide. We show in mice that maternal ID causes severe embryonic cardiovascular defects via premature differentiation of a subset of cardiac progenitor cells. At the molecular level, this most likely results from increased retinoic acid signalling. Furthermore, we show that the defects can be rescued by iron administration early in pregnancy, by reducing vitamin A intake in iron deficient mothers or by treatment with an inhibitor of RA signalling. Although our results do not formally distinguish between ID or generalised anaemia as the cause of the defects, we believe it is more likely to be ID. The evidence for this is three-fold. Firstly, we demonstrate that maternal ID phenocopies embryonic loss of CYP26 activity in the heart, coronary vessels and lymphatic system, phenotypes that in each case result from increased embryonic RA signalling. CYP26 is an iron-dependent enzyme, therefore it is possible that ID could partially reduce its enzymatic activity, resulting in mildly increased RA signalling. Secondly, maternal exposure to chronic mild hypoxia late in gestation, mimicking the effects of maternal anaemia, causes reduced RA signalling in her offspring’s kidney post-natally^82^, that is, the opposite effect to our observations. Lastly, human epidemiological studies suggest that maternal anaemia only increases offspring CHD risk minimally (adjusted odds ratio (OR) 1.2^83–85^), whereas low iron intake in the first trimester (with or without overt anaemia) has an adjusted OR of offspring CHD of up to 5.0^11^. Our hypothesis that ID causes mildly increased RA signalling is supported by studies of the effects of exposure of pregnant mice to excess RA^67–69,86^. Here, administration of low doses of RA results in the same phenotypes as ID at E15.5, including isolated membranous VSD and DORV. By contrast, administration of high doses of RA causes TGA, which we do not observe in ID embryos. Furthermore, low doses of RA also cause highly similar morphological defects to ID earlier in development: shortening and rotational defects of the OFT at E10.5; hypoplasia and dysplasia of the proximal OFT cushions (but not the AVC cushions) and hypoplastic or absent aortic ICC at E12.5;^67–69^ and thin ventricular myocardium at E13.5^86^. However, one feature of our hypothesis that is difficult to explain is why ID has a relatively minor effect on embryogenesis. Iron is required for the function of almost 400 human proteins^87^, and one might imagine that the activities of many of these proteins would also be affected in ID embryos. Why the CYP26 enzymes might be particularly sensitive to reduced iron levels in the embryo remains unclear.

Our observations have important clinical implications. We induce maternal ID in mice via environmental modification. However, maternal ID can also arise from genetic insufficiency and this could potentially have similar effects on embryonic development. The induction of identical phenotypes in different individuals by genetic, environmental or gene–environment interaction is called phenocopying. This has previously been suggested to occur in some types of human congenital abnormalities, for example congenital NAD deficiency disorder^88,89^. Our results suggest that some cases of CHD or craniofacial defects might arise from maternal mutations in the almost 40 genes required for iron transport and/or metabolism. To address how common such mutations might be in humans, we examined the Genome Aggregation Database^90^. This is an aggregate of human exome and genome sequencing data from 141,456 individuals without severe paediatric diseases. We identified 771 predicted loss-of-function variants in these genes. Individuals carrying these variants might be predisposed to developing iron deficiency, and thus may have increased risk of having offspring with birth defects.

Our discoveries could also explain some of the variable penetrance of CHD and cleft palate in children with DS^91^. Our unexpected observation of a strong gene–environment interaction in mice resulting in a failure of secondary palate fusion supports our hypothesis that ID causes increased RA signalling. *Cyp26b1* null mouse embryos have fully penetrant cleft palate^92,93^, and high maternal doses of Vitamin A can also induce cleft palate in mouse^94^. In addition, people with DS have an increased risk of cleft palate^95^. Thus the combination of mildly increased RA due to ID, combined with a genetic susceptibility due to DS, may explain our results. This hypothesis is further supported by the presence of PTA in some *Dp1Tyb* + ID embryos. This phenotype is typical of offspring of pregnant mice administered high doses of RA^67–69^, but is not observed in ID alone or *Dp1Tyb* + control embryos. However, the link between *Dp1Tyb* and RA signalling is not obvious. The duplicated region contains 172 protein coding genes, of which at least three have been associated with RA signalling: *Nrip1*, *Runx1* and *Ripply3*. Intriguingly, Ripply3 is a transcriptional co-repressor of Tbx1^96^ and is a direct target of RA signalling^97^. Thus, in *Dp1Tyb* + ID embryos, slightly increased RA signalling coupled with an extra copy of *Ripply3*, may cause repression of Tbx1 target genes, resulting in a phenocopy of *Tbx1* null phenotypes, including cleft palate^98^ and more severe cardiovascular defects. Finally, the clinical relevance of our gene–environment interaction observations could be relatively easily tested by a retrospective clinical study investigating the phenotypes of children with DS and the peri-conception iron status of their mothers.

Our finding that combined iron and vitamin A deficiency substantially rescues the cardiovascular defects may explain the disparity between animal experiments and epidemiological studies of VAD. There is very strong evidence in animal models that VAD alone is highly teratogenic, but epidemiological studies to date have not found a particularly strong association between VAD and CHD. In the developing world, 15% of pregnant women have VAD^99^ and these women also commonly have ID as well. Our results might suggest that the combination of iron and vitamin A deficiency may balance RA levels, and thus mask any effect of VAD alone on CHD prevalence.

Studies of genetic causes of birth defects are useful on a case-by-case basis to provide information on prognosis, treatment options and recurrence rate. However, understanding particular genetic causes of birth defects has limited value in reducing their overall birth prevalence. By contrast, our elucidation of ID as a potential environmental risk and/or modifying factor for CHD may guide changes in clinical advice. Current WHO and National Institute for Health and Care Excellence (NICE) guidelines have conflicting advice on iron supplementation during pregnancy, with the WHO recommending daily iron supplementation to all pregnant women, whereas NICE recommends supplementation only in cases of substantial ID^100,101^. With the caveat that our results are from work in an animal model and there are other factors in women that would need to be taken into consideration (for example, how infections in women may affect iron levels so care is needed on levels of supplementation and questions on how to monitor “safe” levels of iron in women), our results suggest that women planning pregnancy might be advised to maintain optimal iron levels throughout pregnancy. This conclusion is supported by a recent study suggesting that low iron intake during early pregnancy in humans increases the risk of offspring CHD by up to 5-fold^11^.

## Methods

### Animals

All animal experiments were compliant with the UK Animals (Scientific Procedures) Act 1986 and approved by the University of Oxford animal welfare review board and the Home Office (project license PB01E1FB3). Mice were housed in an SPF facility free from the major rodent pathogens except *Helicobacter hepaticus*, with a 12:12 h light/dark cycle, at 19–23 °C, 55 ± 10% humidity, in individually ventilated cages (Tecniplast UK Ltd, Rushden, UK) containing Grade 4 Aspen Chip bedding (Datesand Ltd, Manchester, UK), cardboard tunnels and Sizzle Pet nesting material (LBS Biotechnology, Horley, UK), with free access to food and tap water. Bedding was changed fortnightly, and animals were assessed daily for welfare. Mice were fed with Teklad 2916 or TD.08713 (control), TD.99397 (iron deficient) or TD.190023 (iron and vitamin A deficient), all from Envigo, Belton, UK. C57BL/6 J mice were purchased from Charles River UK. Genetically modified mouse strains were; *Tg(Mef2c-EGFP)#Krc* (Mef2c-AHF-GFP);^33^ *Tg(RARE-Hspa1b/lacZ)12Jrt* (RARE-LacZ);^48^ *Dp(16Lipi-Zbtb21)1TybEmcf* (*Dp1Tyb*);^81^ and *Tbx1*^*tm1Bld*^ (*Tbx1*^*LacZ*^)^80^. Genotyping of pups and embryos was done by standard methods using primers listed in Supplementary Table 3. All genetically modified mice were from colonies that had been backcrossed for more than 10 generations onto the C57BL/6 J background, with the exception of the RARE-LacZ strain, which was maintained on a CD-1 background. BMS493 (Merck) was dissolved at 10 mM in ethanol, then diluted in corn oil (Merck) and 0.7 or 3.5 mg/kg administered by oral gavage in the morning of E8.5 and E9.5. Pregnant mice were sacrificed humanely, and embryos dissected in 1 x Hanks Balanced Salt Solution + 10 mM EDTA. Whole embryos were photographed using a Leica M80 Stereomicroscope with a Leica DMC4500 digital camera and Leica LAS 4.8.0 software.

### Blood haemoglobin and liver iron content measurement

Blood haemoglobin levels were measured in fresh blood using a HemoCue^®^ Hb 201^+^ according to the manufacturer’s instructions. Values of two separate samples were averaged. Two liver samples were taken per animal at sacrifice and one snap-frozen in liquid nitrogen, and the other fixed overnight in Formalin. Frozen samples were analysed by inductively coupled plasma mass spectrometry (ICP-MS)^102^. Briefly, tissue was digested in 1 ml 70% nitric acid (438073, Sigma) using a Discover® SP-D 80 automated microwave digestion system (CEM). Samples were analysed using a Thermo Finnigan Element 2 Sector-Field ICP-MS. For calibration, spikes of 0, 0.5, 1, 10, 20 and 100 ng/g iron were added to replicates of a selected sample. An external iron standard (ICP- MS-68-A solution; High Purity Standards) was diluted and measured to confirm the validity of the calibration. Rhodium also was spiked onto each blank, standard, and sample as an internal standard at a concentration of 1 ng/g. Concentrations from ICP-MS were normalised to starting tissue weight. Fixed liver samples were paraffin embedded, sectioned, and stained by the DAB-enhanced Perls method^102^. Briefly, slides were immersed for 1 h in 1% potassium ferricyanide in 0.1 M HCl buffer and then stained with DAB chromogen substrate (K3468, Dako). Slides were counterstained with hematoxylin and imaged with a Nikon COOLSCOPE slide scanner and staining intensity was measured by colour deconvolution with the H DAB vector in FIJI 2.0.0-rc-69/1.52p software.

### Embryo and heart morphology assessment

Micromagnetic resonance imaging (µMRI) was performed as previously described^103,104^, using either a Varian 9.4 T VNMRS 20 cm horizontal-bore system (Varian Inc. Palo Alto, CA, USA) or a 11.7 T (500 MHz) vertical magnet (Magnex Scientific, Oxon, UK), both running a Varian/Agilent DDR2 console. Briefly, fixed embryos were incubated in 2 mM Magnevist® (Bayer) and embedded in agarose in a Wilmad LabGlass 28-PP-9” tube. Parameters for the 9.4 T system were: TR 28 ms, TE 16 ms, flip angle 52°, 5 averages, 27 × 27 × 27 mm^3^ with a matrix size of 512^3^, giving an isotropic resolution of 52 × 52 × 52 µm^3^ per voxel. A hard RF pulse (duration 100 ms) was used for excitation and receiver bandwidth of 66 kHz. Parameters for the 11.7 T system were: TR 16.8 ms; TE 4.5 ms; 50° flip angle (BIR-4 adiabatic); 9 averages, 24 × 24 × 32 mm^3^ FOV; 1024 × 1024 × 1366 matrix size, 50 kHz bandwidth, 75% partial Fourier scheme;^105^ and reconstructed with the Berkley advanced reconstruction toolkit v0.6 (https://zenodo.org/record/3934312) as 1536 × 1536 × 2049 (complex double) voxels with an isotropic 15.6 µm^3^ resolution. In all cases, µMRI was followed by paraffin embedding, sectioning and H&E staining. High resolution episcopic microscopy (HREM) was performed as previously described^106^. Briefly, samples were fixed overnight at room temperature in Bouin’s fixative (HT10132, Merck), then washed extensively in PBS, before dehydrated in a graded methanol series. Samples were embedded using a JB-4 kit (Catalogue number 00226-1, Polysciences) by overnight infiltration at 4 °C with a 50:50 mix of methanol:infiltration solution (JB-4 Solution A plus 1.25% (w/v) Benzoyl Peroxide Plasticizer), washed once for 60 min at 4 °C in 100% infiltration solution, then incubated for up to 1 week at 4 °C in fresh infiltration solution. Samples were embedded in infiltration solution plus 6% (v/v) JB-4 solution B to initiate polymerisation, and then analysed using a 3D Optical high resolution episcopic microscopy imaging system (Indigo Scientific). µMRI and HREM data were analysed using OsiriX MD DICOM viewer version 9.0.2 (Pixmeo), Horos 3.3.6 (https://horosproject.org) and Amira for Life & Biomedical Sciences version 2019.4 (Thermo Fisher Scientific).

### RNA-Seq

In total, 1110–4566 GFP-expressing viable cells were sorted from E9.5 embryos carrying the Mef2c-AHF-GFP allele^33^ by FACS using a Beckman Coulter MoFlo AstriosEQ with Summit 6.2.7.16492 software. GFP was detected with the 488 nm laser and a 513/26 band pass filter, and DAPI as a viability dye was detected with the 405 nm laser and a 448/59 band pass filter. Cells were collected into Eppendorf DNA LoBind tubes containing lysis buffer, and RNA isolated using a Qiagen RNeasy Mini kit. RNA quality was assessed using RNA pico chips on an Agilent 2100 Bioanalyzer. Library preparation (SMARTer Ultra Low Input RNA for Illumina Sequencing - HV kit) and sequencing (Illumina HiSeq4000) were performed by the High-Throughput Genomics Group at the Wellcome Trust Centre for Human Genetics. The samples were processed sequenced in two batches. Batch 1 contained three control and five hypoxia samples, and batch 2 contained two repeated control samples with five ID samples.

### RNA-Seq data analysis

QC of the raw sequencing reads was performed using FastQC (https://www.bioinformatics.babraham.ac.uk/projects/fastqc). Reads were aligned to Mus musculus genome (mm10) using the splice-aware algorithm STAR v2.5.3a^107^. Gencode version M12 (Ensembl 87) was used for the annotation of the mouse genome. The DNA sequence of the GFP vector was included in the mm10 reference and its transcript was taken into account during alignment. The R Package Rsubread v1.32.4^108^ was used to assign and quantify the reads corresponding to each genomic feature indicated by the mm10 Gencode transcripts. Reads were assigned to the target that has the largest number of overlapping bases. The minimum fraction of overlapping bases in a read that is required for read assignment was 0.25. Counts per million (CPM) values were calculated for each sample and genes were excluded from the analysis if they did not have a CPM of a least 0.5 in a least 2 libraries. Normalisation was performed using trimmed mean of values (TMM) as implemented in the edgeR v3.24.3 R package^109^ to scale the raw library sizes^110^. Voom transformation was performed to prepare the data for linear modelling using limma v3.38.3^111,112^. Principal Component Analysis and Multidimensional scaling was performed for quality control to investigate sample clustering. Batch correction was performed using the ComBat method^113^ in the sva R v3.30.1 package^114^. Differential expression analysis between control and treated samples was performed using limma v3.38.3^112^. Multiple testing correction was performed on the p-values using the Benjamini-Hochberg false-discovery rate (FDR) procedure with an FDR <5%^115^. Differentially expressed genes were determined using adjusted *p* value < 0.01 and logFC >1 or <−1 and *B* > 1. Unsupervised Hierarchical clustering (Fig. 3b) was performed using the Euclidian distance measure and complete agglomeration method and the pheatmap v1.0.12 R package (https://cran.r-project.org/web/packages/pheatmap/index.html).

### Immunohistochemistry, X-gal staining and RNAScope®

All antibodies used for this study are listed in Supplementary Table 4. Immunohistochemistry on paraffin sections was done as previously described^5^. Briefly, embryos were fixed overnight in 4% paraformaldehyde at 4 °C, paraffin embedded and sectioned in the indicated plane. To minimise inter-slide staining variation, tissue arrays were made by putting single sections from 12–20 different embryos on a single slide, and slides were processed using a Shandon Sequenza® Immunostaining Centre (Thermo Fisher Scientific). For detecting ß-GALACTOSIDASE, the signal was amplified using a peroxidase-conjugated secondary antibody and Alexa Fluor™ 488 Tyramide SuperBoost™ Kit (Thermo Fisher Scientific). For immunohistochemistry on wholemount hearts, tissue was fixed overnight in paraformaldehyde at 4 °C. Hearts were washed three times in phosphate buffered saline plus 0.1% Triton X-100 (PBST), blocked in 5% goat serum in PBST for one hour at 4 °C, then incubated in Armenian hamster anti-CD31 monoclonal antibody in PBST plus 1% bovine serum albumin overnight at 4 °C. Hearts were washed three times in PBST, then incubated in biotinylated goat anti-Armenian hamster secondary antibody for 60 min at 4 °C. Hearts were washed three times in PBST, then incubated in avidin-biotin horseradish peroxidase complex (VECTASTAIN® ABC-HRP Kit PK-4000, Vector Laboratories) 1:50 in PBS for 30 min. Finally, hearts were washed three times in PBST and placed in DAB substrate (Peroxidase substrate kit SK-4100, Vector Laboratories). When staining was complete, the reaction was stopped by washing in MilliQ water. X-gal staining of embryos in wholemount was carried out by standard methods^116^. Briefly, freshly dissected embryos carrying the RARE-LacZ allele were incubated in fix solution (0.2% glutaraldehyde, 0.1 M phosphate buffer pH 7.3, 2 mM MgCl2, 5 mM EGTA) for 10 min a t room temperature, washed twice in wash buffer (0.1 M phosphate buffer pH 7.3, 2 mM MgCl2, 0.1% sodium deoxycholate, 0.02% Nonidet™ P40) and stained in stain solution (wash solution plus 0.2 M sodium chloride, 5 mM potassium ferricyanide, 5.7 mM potassium ferrocyanide, 1.7 mM spermidine, 1 mg/ml 5-Bromo-4-chloro-3-indolyl-β-D-galactopyranoside (XGAL-RO, Merck)) for up to 60 min at room temperature. Staining was stopped by washing in wash buffer, embryos fixed overnight in 4% PFA, and embedded in paraffin prior to sectioning. Sections were imaged using a Zeiss Discovery V8 microscope with Zeiss Zen 2012 (blue) software. RNAScope® with the Mm-Gata4-C1 probe (Catalogue number 417881, Bio-Techne, UK). was performed on paraffin sections using a manual RNAscope^®^ Multiplex Fluorescent Reagent Kit v2 (Catalogue number 323100, Bio-Techne, UK), following the manufacturer’s instructions. The *Gata4* signal was developed using Opal™ dye 690 (FP1497001KT, Akoya Biosciences, USA) at 1:750 dilution. After development, slides were processed for immunohistochemistry with the anti-MF20 antibody and stained with DAPI. Slides were imaged variously on: (i) an Olympus FV1000 or FV3000 confocal microscope with Fluoview FV31S-SW software; (ii) a Zeiss LSM980 confocal microscope with Zen Blue 3.3 software.

### Statistical analyses

All statistical analyses were performed with Prism 8.4.2 (GraphPad Software). Data were first tested for normal distribution by Shapiro–Wilk test and equal variance by F test. For data with two groups, normally distributed samples were tested for statistical significance using two-tailed Student’s t test (if variances equal) or two-tailed Welch’s corrected *t* test (if unequal variances). Non-normally distributed samples were tested using a two-tailed Mann–Whitney *U* test. For data with more than two groups, normally distributed data were tested using ANOVA followed by Tukey’s or Dunnett’s post hoc test adjusted for multiple comparisons to compare the means of each group with every other group. Non-normally distributed data were tested using Kruskal–Wallis one-way ANOVA with Dunnett’s post hoc test adjusted for multiple comparisons. The statistical significance of binomial prevalence data was tested using one-tailed Fisher’s exact test. Data are presented as mean ± standard deviation.

### Reporting summary

Further information on research design is available in the Nature Research Reporting Summary linked to this article.

## Supplementary information

Supplementary information_new

Reporting Summary

## Data Availability

The RNA-Seq data supporting the findings of this study have been deposited in the Sequence Read Archive (SRA) database under accession code: BioProject ID PRJNA596545. Source data are provided with this paper. Other data that support the findings of this study are available from the corresponding author upon reasonable request. Source data are provided with this paper.

## Source data

Source data
