# Supplementary material for: Maternal iron deficiency perturbs embryonic cardiovascular development in mice
Source: Nat Commun. 2021 Jun 8;12:3447. doi: 10.1038/s41467-021-23660-5 (PMC8187484; doi:10.1038/s41467-021-23660-5)
Supplement: Supplementary file 1 — Supplementary information_new [file 41467_2021_23660_MOESM1_ESM.pdf]

Supplementary Figure 1. Mice and embryos continuously fed a low iron diet from weaning are ID and anaemic

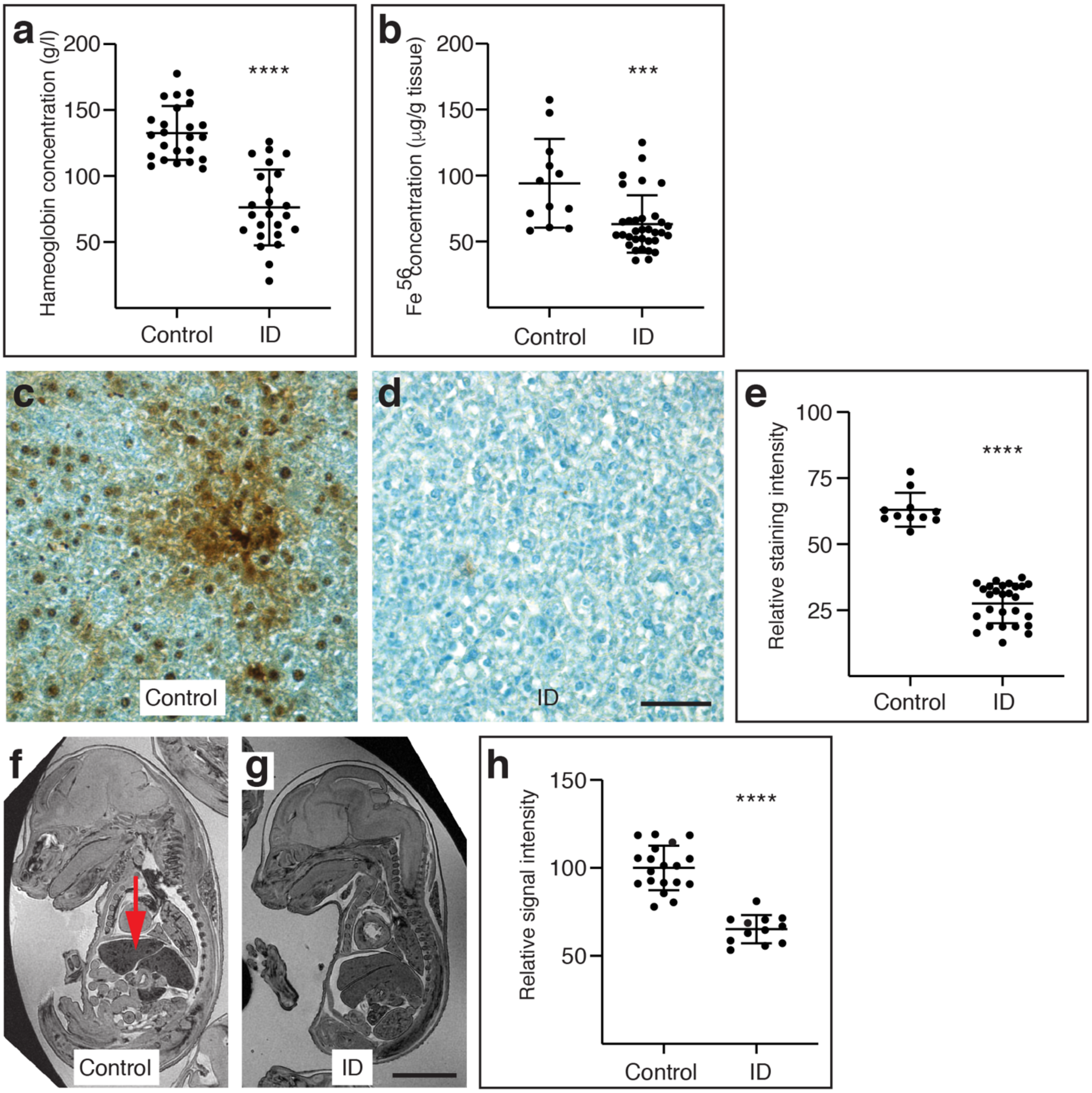

(a) Comparison of blood haemoglobin concentrations between adult female mice fed since weaning on control (200 ppm iron, n=24) or low iron (2-6 ppm iron, n=24) diet. (b) Comparison of Fe^{56} concentration in liver samples measured by Inductively Coupled Plasma Mass Spectrometry from control (n=12) and ID (n=33) mice. (c-e) Representative images showing relative DAB-enhanced Perls staining of liver sections from control (c) or ID (d) mice. (e) Quantitation of Perls staining from livers from control (n=11) and ID (n=27) mice. (f-h) Representative images showing relative liver MRI contrast from control (f) or ID (g) E15.5 embryos. The embryonic liver is shown by a red arrow. (g) Quantitation of liver MRI contrast in control (n=20) and ID (n=12) embryos. Graphs show mean ± standard deviation. Statistical significance was tested by two-sided unpaired t test (panels a,h) or two-sided Mann Whitney U test (panels b,e). P values: <0.0001 (panel a); 0.0005 (panel b); <0.0001 (panel e); <0.0001 (panel h). *** P<0.001, **** P<0.0001. Scale bars are 50 µm (c,d) and 3 mm (f,g).

Supplementary Figure 2. Maternal ID causes cardiovascular defects

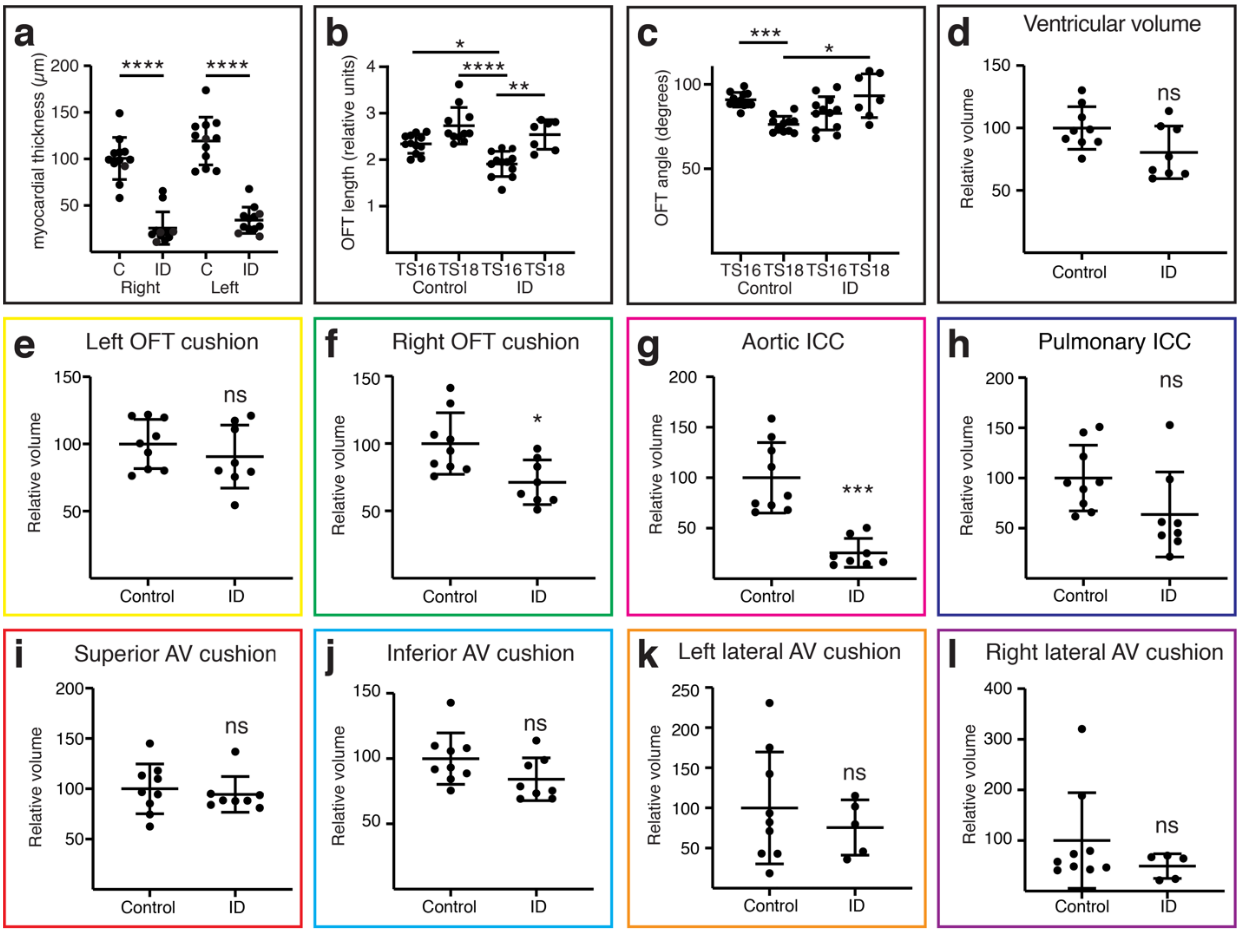

(a) Quantification of the ventricular myocardial thickness of hearts from E15.5 control (n=12) and ID (n=12) embryos. (b-c) Quantification of length (b) and angle between distal and proximal portions of the OFT (c) from control (TS16 n=12, TS18 n=12) and ID (TS16 n=11, TS18 n=7) E10.5 embryos. (d-l) Quantitation of whole ventricle and cardiac cushion volumes from 3D Amira models of manually-segmented HREM data from control (n=9) and ID (n=8, except for lateral AV cushions where n=5) E12.5 embryos. The panel border colour corresponds to the cushion colour in Figure 1 (panels h-q). Graphs show mean ± standard deviation. Statistical significance was tested by two-sided Mann Whitney U test (panels a,i,l), two-sided unpaired t test (panels a',d,e,f,h,j,k), Kruskall-Wallis with Dunn's multiple comparisons test (panels b,c) or two-sided unpaired t test with Welch's correction (panel g) or. P values: <0.0001 (panel a); <0.0001 (panel a'), 0.0295, <0.0001, 0.0036 (panel b); 0.0008, 0.0109 (panel c); 0.0521 (panels d); 0.3679 (panel e); 0.0101 (panel f); 0.0001 (panel g); 0.0650 (panel h); 0.4807 (panel i); 0.0945 (panel j); 0.4820 (panel k); 0.3636 (panel l). ns, not significant, * P<0.05, ** P<0.01, *** P<0.001, **** P<0.0001.

Supplementary Figure 3. Effects of ID on cardiac progenitor proliferation and TBX5 expression

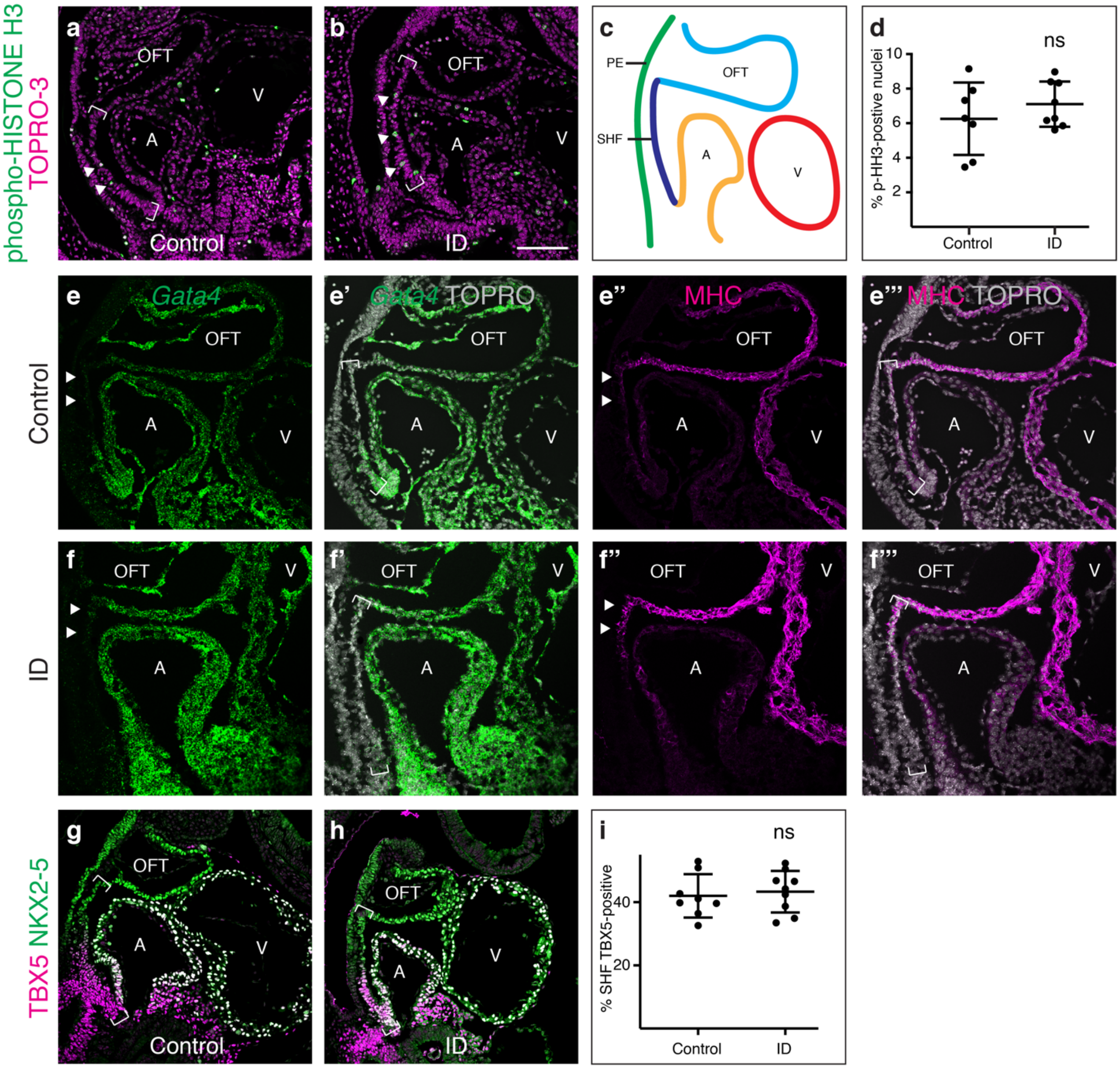

(a-d) Comparison of expression levels of phospho-Histone H3 (pHH3, green) in sagittal sections of control (a) and ID (b) E9.5 mouse embryos by immunohistochemistry. Nuclei were stained with TO-PRO-3 (magenta). Location of the SHF is indicated by brackets. White arrowheads indicate pHH3-positive cells. (c) Diagram indicating the relative positions of the SHF (dark blue), pharyngeal endoderm (green), OFT (light blue) and left ventricle (V, red) and left atrium (A, orange) in a sagittal section of an E9.5 embryo. (d) Quantification of number of pHH3-positive SHF cells in control (n=7) and ID (n=8) embryos. (e-f) Comparison of the expression pattern of *Gata4* transcripts by RNAScope (green) and MHC (magenta) by immunohistochemistry in control (e, n=11) and ID (f, n=7) E9.5 mouse embryos. Nuclei were stained with TO-PRO-3 (gray). Location of the SHF is indicated by brackets. White arrowheads indicate the aSHF. (g-i) Comparison of expression levels of TBX5 (magenta) and NKX2-5 (green) in control (h) and ID (i) E9.5 mouse embryos by immunohistochemistry. Location of the SHF is indicated by brackets. (j) Quantification of the percentage of TBX5-positive SHF in control (n=8) and ID (n=9) embryos. Graphs show mean ± standard deviation. Statistical significance was tested by two-sided unpaired t test (panels d,i). P values: 0.3586 (panel d); 0.6907 (panel i). ns, not significant. Scale bar = 130 µm (a,b,g,h) and 85 µm (e,f).

Supplementary Figure 4. The first intron of the Gata4 gene contains a putative retinoic acid responsive enhancer

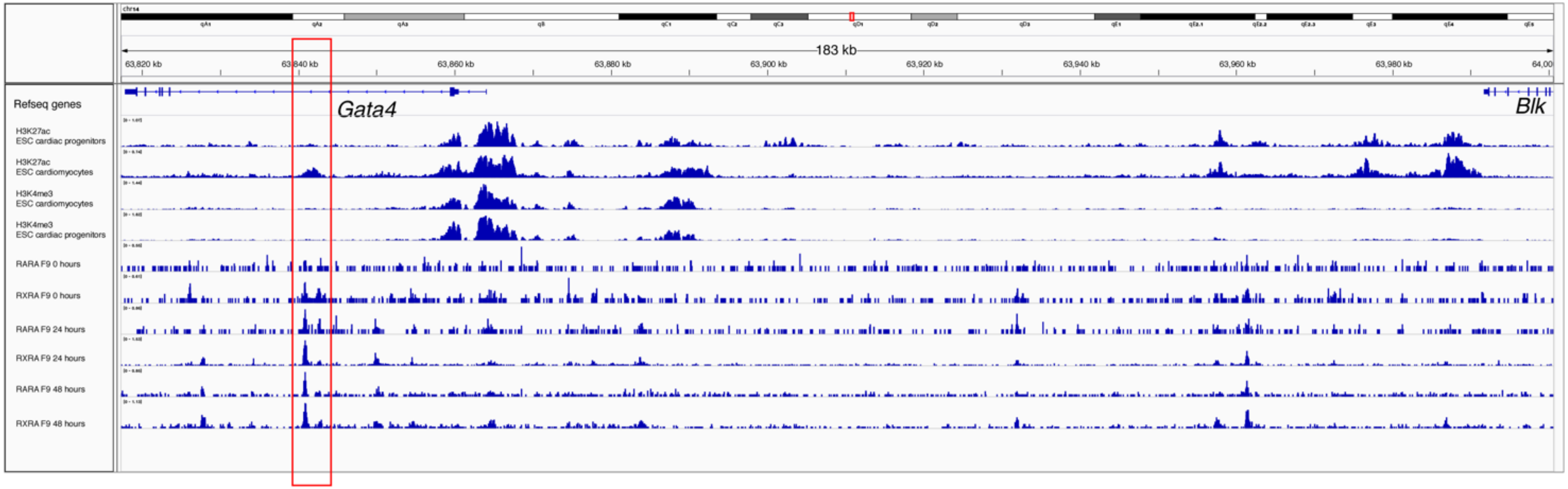

The 5' upstream region and first intron of the mouse *Gata4* gene as seen on the UCSC Browser (http://genome.ucsc.edu). The first four tracks show the show the enhancer-associated H3K27Ac and promoter-associated H3K4Me3 marks found in mouse embryonic stem cells differentiated into cardiac progenitors and cardiomyocytes[1]. The final six tracks show binding data of the retinoic acid receptors RARA and RXRA in F9 embryo carcinoma cells treated with retinoic acid for the indicated times[2]. The cardiomyocyte-specific H3K27Ac histone marks in the first intron of the *Gata4* gene (red box) correlate with RARA and RXRA binding sites in F9 EC cells treated with retinoic acid.

Supplementary Figure 5. Effects of ID on lymphatic vasculature development and skeletal patterning

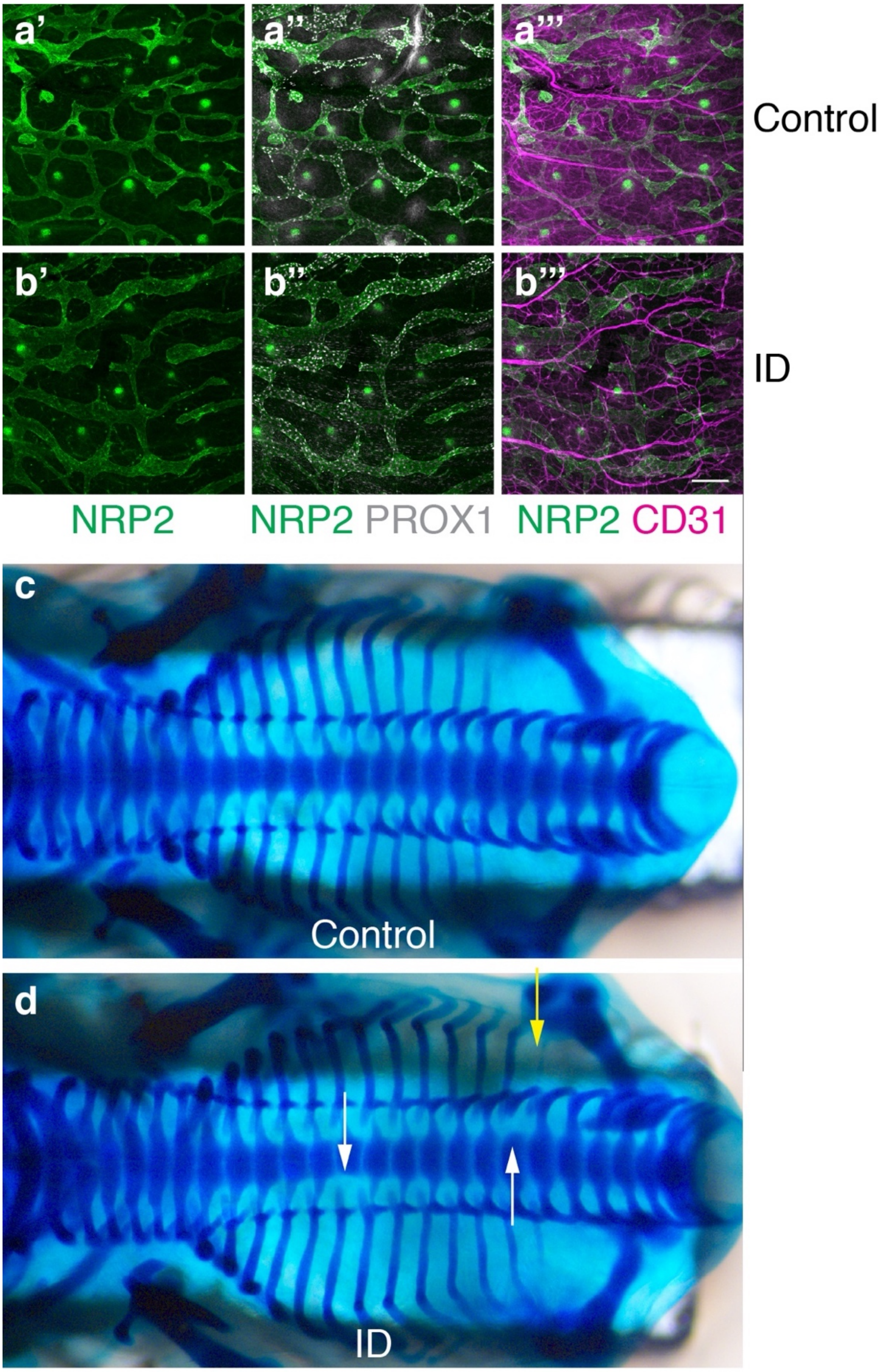

Comparison of NRP2, PROX1 and CD31 expression in back skin from control (n=10) and ID (n=11) E14.5 embryos. Magnified views of the boxed areas from Figure 5a,b are shown in panels a'-a''' and b'-b''', respectively. (c,d) Alcian blue staining of cartilage in control (c, 21/21 embryos) and ID (d, 29/33 embryos) E14.5 embryos. Missing pedicles (black arrows) and rib (yellow arrow) are indicated. Scale bar = 370 μm (a,b) and 260 μm (c,d).

Supplementary Figure 6. Effects of maternal diet on blood haemoglobin levels and embryonic liver iron

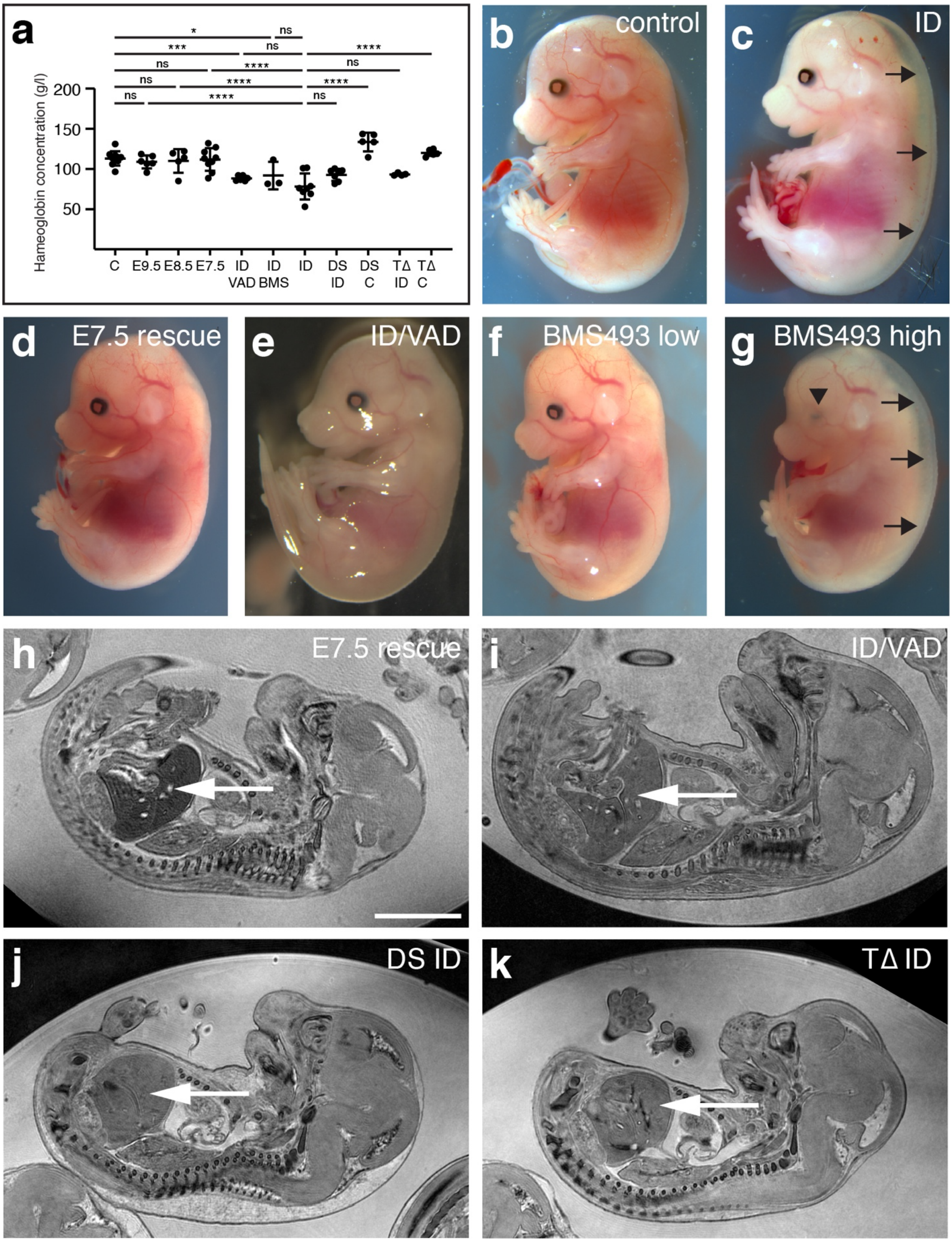

(a) Blood haemoglobin concentrations in pregnant adult C57BL/6J strain female mice measured at E15.5. C, mice fed control diet (n=10); E9.5 (n=6), E8.5 (n=5), E7.5 (n=9), mice fed from weaning on a low iron diet, then returned to control diet at the indicated stage of pregnancy; ID/VAD (n=6), mice fed from weaning on a low iron and Vitamin A-

deficient diet; ID/BMS (n=3), mice fed from weaning on a low iron and treated with 0.7 mg/kg BMS493 on E8.5 and E9.5; ID (n=8), mice fed from weaning on a low iron diet; DS ID (n=7), ID females crossed to *Dp1Tyb*+ males; DS C (n=5), control females crossed to *Dp1Tyb*+ males; TΔ ID (n=4), ID females crossed to $Tbx1^{+/null}$ males; TΔ C (n=7), control females crossed to $Tbx1^{+/null}$ males. (b-g) Representative images of E15.5 embryos from control (b); ID (c); (d) ID mothers returned to control diet on E7.5; (e) mothers fed a low iron and vitamin A-deficient diet; (f) ID mothers treated with 0.7 mg/kg BMS493; and (g) ID mothers treated with 3.5 mg/kg BMS493. Subcutaneous oedema (black arrows) and coloboma (black arrowhead) are indicated. Panels b and c are repeated from Figure 1. (h-k) Representative images showing relative liver MRI contrast in E15.5 embryos from (h) ID mothers returned to control diet on E7.5; (i) mothers fed a low iron and vitamin A-deficient diet; (j) *Dp1Tyb*+ embryos from ID mothers; (k) $Tbx1^{+/null}$ embryos from an ID mother. The embryonic livers are shown by a white arrow. Graphs show mean ± standard deviation. Statistical significance was tested using one way ANOVA with Dunnett's multiple comparison test. P values: 0.9913 (C vs E9.5); 0.9993 (C vs E8.5); 0.9997 (C vs E7.5); 0.0005 (C vs ID VAD); 0.0 376 (C vs ID BMS); <0.0001 (ID vs E9.5); <0.0001 (ID vs E8.5); <0.0001 (ID vs E7.5); 0.5029 (ID vs ID VAD); 0.4063 (ID vs ID BMS); 0.0953 (ID vs DS ID); <0.0001 (ID vs DS C); 0.1945 (ID vs TΔ ID); <0.0001 (ID vs TΔ C). ns, not significant, * P<0.05, *** P<0.001, **** P<0.0001. Scale bar = 4.4 mm (b-g) and 3 mm (h-k).

Supplementary Table 1. Summary of types of heart defects observed in E15.5 embryos.
ASD, atrial septal defect; mVSD: membranous ventricular septal defect; musVSD: muscular ventricular septal defect; AVSD, atrioventricular septal defect; OFT, outflow tract defects (includes overriding aorta and double-outlet right ventricle); PTA, persistent *truncus arteriosus*. † one with type I PTA; ‡ three with type I PTA. Statistical significance was tested using one-sided Fisher's exact test.

Types of defects		Control	ID	E7.5 rescue	ID VAD	ID BMS4 93		*Dp1Tyb*+ ID	wild type ID	*Dp1Tyb*+ control		*Tbx1*$^{+/null}$ ID	*Tbx1*$^{+/+}$ ID	*Tbx1*$^{+/null}$ control
Single	ASD	0	0	0	0	0		0	0	0		0	0	0
	mVSD	1	5	0	0	0		0	1	0		1	1	0
	muscVSD	0	11	0	0	0		3	1	1		4	3	0
	AVSD	0	0	0	0	0		1	0	0		0	0	0
Multiple	OFT + mVSD	0	1	0	2	1		0	2	0		1	0	0
	mVSD + muscVSD	0	4	0	0	0		0	1	0		2	1	0
	OFT + mVSD + muscVSD	0	4	0	0	0		3†	2	0		4	1	1
	mVSD + AVSD	0	0	0	0	0		0	0	0		0	0	0
	OFT + mVSD + AVSD	0	0	0	0	0		1	1	0		0	0	0
	muscVSD + AVSD	0	4	0	0	0		0	0	0		0	1	0
	OFT + muscVSD + AVSD	0	1	0	1	0		8‡	2	0		1	2	0
Total abnormal		1	30	0	3	1		16	10	1		13	9	1
Total normal		36	12	26	22	11		2	9	15		3	3	18

P value vs control		-	<0.0001	0.5873	0.1752	0.4337	P value vs *Dp1Tyb* + control	<0.0001	0.0037	-	P value vs *Tbx1*$^{+/null}$ control	<0.0001	<0.0001	-
P value vs ID		-	-	<0.0001	<0.0001	0.0001	P value vs wild type	0.0186	-	-	P value vs *Tbx1*$^{+/+}$ ID	0.5208	-	-

Supplementary Table 2. Summary of aortic arch morphology at E15.5

IAA, interrupted aortic arch; A-RSA, aberrant right subclavian artery; R-LCC, retroesophageal left subclavian artery.
Note that an embryo may have more than one phenotype. Statistical significance was tested using one-sided Fisher’s exact test

Types of defects	control	ID	E7.5 rescue	ID VAD	ID BMS493		*Dp1Tyb+* ID	wild type ID	*Dp1Tyb+* control		*Tbx1*$^{+/null}$ ID	*Tbx1*$^{+/+}$ ID	*Tbx1*$^{+/null}$ control
IAA	0	3	0	0	0		4	1	0		0	0	1
A-RSA	0	1	1	1	0		1	3	0		4	1	4
R-LCC	0	1	0	0	0		2	0	1		0	0	0
right-sided AA	1	1	0	0	0		2	0	1		2	0	1
abnormal	1	5	1	1	0		7	4	1		6	1	6
normal	36	35	24	24	11		11	15	14		10	11	13
P value vs control	-	0.1189	0.6478	0.6478	0.7708	P value vs *Dp1Tyb+* control	0.0375	0.2507	-	P value vs *Tbx1*$^{+/null}$ control	0.4946	0.1430	-
P value vs ID	-	-	0.2456	0.2456	0.2801	P value vs wild type ID	0.2046	-	-	P value vs *Tbx1*$^{+/+}$ ID	0.0908	-	-

Supplementary table 3

Genotyping primer sequences

Strain	Sequence	Product
Tg(Mef2c-EGFP)#Krc	GTGAGGAGGGAGCTGCAGTATGTTTG	550 bp
	AAGTCGTGCTGCTTCATGTG	
Tg(RARE-Hspa1b/lacZ)12Jrt	ATCCTCTGCATGGTCAGGTC	300 bp
	CGTGGCCTGATTCATTCC	
Dp(16Lipi-Zbtb21)1TybEmcf	CCTCGAGCTGTTCCTGCTAT	500 bp
	GAAGAATTTCTGTGGGGCAA	
Tbx1^{tm1Bld}	CTGGGCACAACAGACAATCGGCT	500 bp
	TATTCGGCAAGCAGGCATCGCCA	

Supplementary Table 4

Antibodies

**Target**	**Name**	**Catalog number**	**Species and type**	**Supplier**	**Dilution**
**Primary antibodies**					
MYOSIN II HEAVY CHAIN	MF 20		mouse monoclonal	Developmental Studies Hybridoma Bank[3]	1:100
phospho-HISTONE H3	(Ser 10)-R	sc-8656	rabbit polyclonal	Santa Cruz Biotechnology	1:200
GATA4		sc-25310	mouse monoclonal	Santa Cruz Biotechnology	1:100
TBX5		sc-515536	mouse polyclonal	Santa Cruz Biotechnology	1:500
NKX2-5	N-19	sc-8697	goat polyclonal	Santa Cruz Biotechnology	1:250
CD31 (heart)		ab119341	Armenian hamster monoclonal	Abcam	1:500
CD31 (back skin)		553370	rat monoclonal	BD Pharmingen	1:500
PROX1		AF2727	goat polyclonal	R&D systems	1:500
NRP2	D39A5	3366	rabbit monoclonal	Cell Signaling Technology	1:500
ß-GALACTOSIDASE		ab9361	Chicken polyclonal	Abcam	1:200
**Secondary antibodies**					
Donkey anti-mouse Cy™3		715-165-151		Jackson ImmunoResearch	1:500
Donkey anti-mouse AlexaFluor® 488		715-545-151		Jackson ImmunoResearch	1:500
Donkey anti-rabbit Cy™3		711-165-152		Jackson ImmunoResearch	1:500
Donkey anti-rabbit AlexaFluor® 488		711-545-152		Jackson ImmunoResearch	1:500
Donkey anti-goat AlexaFluor® 488		A-11055		Thermo Fisher Scientific	1:500
Donkey anti-rat Dylight™ 405		712-475-153		Jackson ImmunoResearch	1:500
Goat anti-Armenian hamster Biotinylated		ab5744		Abcam	1:250
Donkey anti-chicken IgY Peroxidase		703-035-155		Jackson ImmunoResearch	1:500
**Nuclear stain**					
TO-PRO®-3 Iodide		T3605		Life Technologies	1:10,000

Supplementary References

1. Wamstad, J. A. *et al.* Dynamic and coordinated epigenetic regulation of developmental transitions in the cardiac lineage. *Cell* **151**, 206-220, doi:10.1016/j.cell.2012.07.035 (2012).
2. Chatagnon, A. *et al.* RAR/RXR binding dynamics distinguish pluripotency from differentiation associated cis-regulatory elements. *Nucleic Acids Res* **43**, 4833-4854, doi:10.1093/nar/gkv370 (2015).
3. Bader, D., Masaki, T. & Fischman, D. A. Immunochemical analysis of myosin heavy chain during avian myogenesis in vivo and in vitro. *J Cell Biol* 95, 763-770, doi:10.1083/jcb.95.3.763 (1982).
