# Supplementary material for: Maternal iron deficiency perturbs embryonic cardiovascular development in mice
Source: Nat Commun. 2021 Jun 8;12:3447. doi: 10.1038/s41467-021-23660-5 (PMC8187484; doi:10.1038/s41467-021-23660-5)
Supplement: Supplementary file 2 — Reporting Summary [file 41467_2021_23660_MOESM2_ESM.pdf]

nature research

Corresponding author(s): Duncan B Sparrow

Last updated by author(s): Apr 28, 2021

Reporting Summary

Nature Research wishes to improve the reproducibility of the work that we publish. This form provides structure for consistency and transparency in reporting. For further information on Nature Research policies, see our Editorial Policies and the Editorial Policy Checklist.

Statistics

For all statistical analyses, confirm that the following items are present in the figure legend, table legend, main text, or Methods section.

n/a	Confirmed	
☐	☒	The exact sample size (n) for each experimental group/condition, given as a discrete number and unit of measurement
☐	☒	A statement on whether measurements were taken from distinct samples or whether the same sample was measured repeatedly
☐	☒	The statistical test(s) used AND whether they are one- or two-sided<br>*Only common tests should be described solely by name; describe more complex techniques in the Methods section.*
☒	☐	A description of all covariates tested
☐	☒	A description of any assumptions or corrections, such as tests of normality and adjustment for multiple comparisons
☐	☒	A full description of the statistical parameters including central tendency (e.g. means) or other basic estimates (e.g. regression coefficient) AND variation (e.g. standard deviation) or associated estimates of uncertainty (e.g. confidence intervals)
☐	☒	For null hypothesis testing, the test statistic (e.g. F, t, r) with confidence intervals, effect sizes, degrees of freedom and P value noted<br>*Give P values as exact values whenever suitable.*
☒	☐	For Bayesian analysis, information on the choice of priors and Markov chain Monte Carlo settings
☒	☐	For hierarchical and complex designs, identification of the appropriate level for tests and full reporting of outcomes
☒	☐	Estimates of effect sizes (e.g. Cohen's d, Pearson's r), indicating how they were calculated

Our web collection on statistics for biologists contains articles on many of the points above.

Software and code

Policy information about availability of computer code

Data collection	Image data was collected using: Leica LAS 4.8.0; Olympus Fluoview FV31S-SW; Zeiss Zen 2012 (blue); and Zeiss Zen Blue 3.3.<br>Custom code for µMRI was as described in Cleary, J. O. et al. NMR Biomed 22, 857-866, doi:10.1002/nbm.1400 (2009); Zamyadi, M. et al. Physiol Genomics 42A, 89-95, doi:10.1152/physiolgenomics.00091.2010 (2010); and Margosian, P., DeMeester, G. & Liu, H. in eMagRes (eds R.K Harris & R.L. Wasylishen). doi: 0.1002/9780470034590.emrstm0376 (2007). µMRI data was reconstructed with the Berkley advanced reconstruction toolkit v0.6 (https://zenodo.org/record/3934312).
Data analysis	HREM and µMRI: Osirix 9.0.2; Horos 3.3.6; Thermo Scientific Amira 2019.4.<br>Image analysis: FIJI 2.0.0-rc-69/1.52p.<br>FACS: Beckman Coulter Summit 6.2.7.16492.<br>Statistical analysis: Prism 8.4.2.<br>RNASeq<br>Read alignment: STAR v2.5.3a. Read assignment and quantification: Rsubread v1.32.4 R Package. Normalisation: edgeR v3.24.3 R package. Voom transformation and differential expression analysis: limma v3.38.3. Batch correction: ComBat in the sva R v3.30.1 package. Unsupervised Hierarchical clustering: pheatmap v1.0.12 R package.

For manuscripts utilizing custom algorithms or software that are central to the research but not yet described in published literature, software must be made available to editors and reviewers. We strongly encourage code deposition in a community repository (e.g. GitHub). See the Nature Research guidelines for submitting code & software for further information.

Data

Policy information about availability of data

All manuscripts must include a data availability statement. This statement should provide the following information, where applicable:

- Accession codes, unique identifiers, or web links for publicly available datasets
- A list of figures that have associated raw data
- A description of any restrictions on data availability

The RNA-Seq data supporting the findings of this study have been deposited in the Sequence Read Archive (SRA) with BioProject ID PRJNA596545. Source data are provided with this paper. Other data that support the findings of this study are available from the corresponding author upon reasonable request.

Field-specific reporting

Please select the one below that is the best fit for your research. If you are not sure, read the appropriate sections before making your selection.

- [x] Life sciences
- [ ] Behavioural & social sciences
- [ ] Ecological, evolutionary & environmental sciences

For a reference copy of the document with all sections, see nature.com/documents/nr-reporting-summary-flat.pdf

Life sciences study design

All studies must disclose on these points even when the disclosure is negative.

Sample size	Sample size was predetermined using online software (powerandsamplesize.com). Separate calculations were used for experiments with binomial output (e.g. qualitative studies such as morphology assessment) or continuous variables (e.g. quantitative experiments such as measurement of blood haemoglobin levels). Sample sizes were calculated to obtain 90% power with a 5% significance level, and were on historical and preliminary data to determine approximate phenotype proportions (for binomial data) or means and standard deviations (for continuous data).
Data exclusions	No data were excluded from the analyses.
Replication	Adult animals.<br>Blood haemoglobin: two measurements were made from each animal.<br>Liver histology and liver iron content: measurements were taken from two sections per liver.<br>All attempts at replication were successful<br><br>Embryos.<br>Embryos for analysis were collected from at least 3 independent litters per experiment. All litters contained embryos with a similar range of cardiovascular defects.<br>Immunohistochemistry; RNAScope and X-gal staining: where possible, a minimum of 3 sections from each embryo were measured and/or scored for morphology. All attempts at replication were successful.<br>Lymphatic vessels: measurements were taken from 5 regions of back skin per embryo. All attempts at replication were successful.<br>Embryo morphology: morphology was scored independently by two researchers.<br>RNASeq: 3-5 independent samples were analysed per experimental group.
Randomization	Wild type female mice were randomly allocated to experimental groups fed a modified or a control diet. Wild type males or genetically-altered males of the required genotype(s) were randomly selected for each timed mating. Embryos of the required developmental stage resulting from timed matings were randomly assigned to smaller groups for analysis for each separate technique.
Blinding	During embryo collection in experiments on wild type mice, blinding was not possible. This is because all embryos in a particular litter are exposed to the same environmental conditions. Embryo collection in experiments on genetically-altered mice were blinded for embryo genotype. After collection, all data analysis was done by a researcher blinded to group assignment.<br>For experiments not involving embryos (measurement of maternal iron status), researchers were not blinded to group assignment for data collection. This is because the phenotype of anaemic mice was easily distinguishable from controls. After collection, all data analysis was done by a researcher blinded to group assignment.

Reporting for specific materials, systems and methods

We require information from authors about some types of materials, experimental systems and methods used in many studies. Here, indicate whether each material, system or method listed is relevant to your study. If you are not sure if a list item applies to your research, read the appropriate section before selecting a response.

Materials & experimental systems

n/a	Involved in the study	
☐	☒	Antibodies
☒	☐	Eukaryotic cell lines
☒	☐	Palaeontology and archaeology
☐	☒	Animals and other organisms
☒	☐	Human research participants
☒	☐	Clinical data
☒	☐	Dual use research of concern

Methods

n/a	Involved in the study	
☒	☐	ChIP-seq
☒	☐	Flow cytometry
☒	☐	MRI-based neuroimaging

Antibodies

Antibodies used

MYOSIN II HEAVY CHAIN, MF 20, mouse monoclonal, Developmental Studies Hybridoma Bank.
phospho-HISTONE H3 (Ser 10)-R, sc-8656, rabbit polyclonal, Santa Cruz Biotechnology, lot B0516.
GATA4, sc-25310, mouse monoclonal, Santa Cruz Biotechnology, lot A1218.
TBX5, sc-515536, mouse polyclonal, Santa Cruz Biotechnology, lot G1516.
NKX2-5 N-19, sc-8697, goat polyclonal, Santa Cruz Biotechnology , lot L2215.
CD31, ab119341, Armenian hamster monoclonal, Abcam, lot GR3216937-3.
CD31, 553370 rat monoclonal BD Pharmingen, lot 7257819.
PROX1, AF2727, goat polyclonal, R&D systems, lot VIY0217121.
NRP2 D39A5, 3366, rabbit monoclonal, Cell Signaling Technology, lot 2.
ß-GALACTOSIDASE, ab9361, chicken polyclonal, Abcam, lot GR323668-14.
anti-mouse Cy™3, 715-165-151, Donkey polyclonal, Jackson ImmunoResearch, lot 128010.
anti-mouse AlexaFluor® 488, 715-545-151, Donkey polyclonal, Jackson ImmunoResearch, lot 127820.
anti-rabbit Cy™3, 711-165-152, Donkey polyclonal, Jackson ImmunoResearch, lot 127720.
anti-rabbit AlexaFluor® 488, 711-545-152, Donkey polyclonal, Jackson ImmunoResearch, lot 133590.
anti-goat AlexaFluor® 488, A-11055, Donkey polyclonal, Thermo Fisher Scientific, lot 1771339.
anti-rat Dylight™ 405, 712-475-153, Donkey polyclonal, Jackson ImmunoResearch, lot 133804.
anti-Armenian hamster Biotinylated, ab5744, Goat polyclonal, Abcam.
anti-chicken IgY Peroxidase, 703-035-155, Donkey polyclonal, Jackson ImmunoResearch, lot 149885.

Validation

Optimal Ab concentration was titrated in-house by immunofluorescence and immunohistochemistry. Further validation information of the antibodies by the manufacturer is as follows:
Note: for further information where available, Antibody Registry (antibodyregistry.org) details are given (AB_####).
Anti-MYOSIN II HEAVY CHAIN MF 20 mouse monoclonal antibody from the Developmental Studies Hybridoma Bank (AB_2147781). Many published validations listed on website (dshb.biology.uiowa.edu/MF-20); anti-phospho-HISTONE H3 sc-8656 rabbit polyclonal antibody from Santa Cruz Biotechnology (AB_653256, now discontinued). Several published validations on website (www.scbt.com/p/p-histone-h3-antibody-ser-10); Anti-GATA4 sc-25310 mouse monoclonal antibody from Santa Cruz Biotechnology (AB_627667). Immunofluoresence validation shown on website (https://www.scbt.com/p/gata-4-antibody-g-4); Anti-TBX5 sc-515536 mouse polyclonal antibody from Santa Cruz Biotechnology. Validation shown on website (https://www.scbt.com/p/tbx5-antibody-a-6). Anti-NKX2-5 N-19 sc-8697 goat polyclonal antibody from Santa Cruz Biotechnology (AB_650280, now discontinued). Many published validations on website (https://www.scbt.com/p/nkx-2-5-antibody-n-19); Anti-CD31 ab119341 Armenian hamster monoclonal antibody from Abcam (AB_10900179). Immunohistochemistry validation shown on website (https://www.abcam.com/cd31-antibody-2h8-ab119341.html); Anti-CD31 553370 rat monoclonal antibody from BD Pharmingen (AB_394816). Many published validations on website (https://www.bdbiosciences.com/eu/applications/research/stem-cell-research/cancer-research/mouse/purified-rat-anti-mouse-cd31-mec-133/p/553370); Anti-PROX1 AF2727 goat polyclonal antibody from R&D systems (AB_2170716). Immunofluorescence validation shown on website (https://www.rndsystems.com/products/human-prox1-antibody_af2727); Anti-NRP2 D39A5 3366 rabbit monoclonal antibody from Cell Signaling Technology (AB_2155250). Immunofluorescence validation shown on website (https://www.cellsignal.co.uk/products/primary-antibodies/neuropilin-2-d39a5-xp-rabbit-mab/3366). Anti-ß-GALACTOSIDASE ab9361 chicken polyclonal from Abcam (AB_307210). Immunofluorescence validation shown on website (https://www.abcam.com/beta-galactosidase-antibody-ab9361.html). anti-mouse Cy™3 715-165-151 Donkey polyclonal antibody from Jackson ImmunoResearch (AB_2315777). Validation shown on website (https://www.jacksonimmuno.com/catalog/products/715-165-151). anti-mouse AlexaFluor® 488 715-545-151 Donkey polyclonal antibody from Jackson ImmunoResearch (AB_2341099). Validation shown on website (https://www.jacksonimmuno.com/catalog/products/715-545-151). anti-rabbit Cy™3 711-165-152 Donkey polyclonal antibody from Jackson ImmunoResearch (AB_2307443). Validation shown on website (https://www.jacksonimmuno.com/catalog/products/711-165-152). anti-rabbit AlexaFluor® 488 711-545-152 Donkey polyclonal antibody from Jackson ImmunoResearch (AB_2313584). Validation shown on website (https://www.jacksonimmuno.com/catalog/products/711-545-152). anti-goat AlexaFluor® 488 A-11055 Donkey polyclonal antibody from Thermo Fisher Scientific (AB_2534102). Validation shown on website (https://www.thermofisher.com/antibody/product/A-11055.html?CID=AFLCA-A-11055).
anti-rat Dylight™ 405 712-475-153 Donkey polyclonal antibody from Jackson ImmunoResearch (AB_2340681). Validation shown on website (https://www.jacksonimmuno.com/catalog/products/712-475-153). anti-Armenian hamster Biotinylated, ab5744 Goat polyclonal antibody from Abcam (AB_954919). Validation shown on website (https://www.abcam.com/goat-armenian-hamster-igg-hl-biotin-ab5744.html). anti-chicken IgY Peroxidase 703-035-155 Donkey polyclonal antibody from Jackson ImmunoResearch (AB_10015283). Validation shown on website (https://www.jacksonimmuno.com/catalog/products/703-035-155).

Animals and other organisms

Policy information about studies involving animals; ARRIVE guidelines recommended for reporting animal research

Laboratory animals	Strain information<br>Species: Mouse<br>Strains:<br>C57BL/6J<br>Tg(Mef2c-EGFP)#Krc<br>Dp(16Lipi-Zbtb21)1TybEmcf<br>Tbx1tm1Bld<br>Tg(RARE-Hspa1b/lacZ)12Jrt<br>Genetic background: All genetically-modified mice were from colonies that had been backcrossed for more than 10 generations onto the C57BL/6J background, with the exception of the Tg(RARE-Hspa1b/lacZ)12Jrt strain, which was maintained on a CD-1 background.<br>Sex: Adult males from C57BL/6J and all four genetically-altered mouse strains were mated with female adult C57BL/6J mice. Data was collected and analysed from adult female mice, and both male and female embryos.<br>Age: Females 8-26 weeks; Males (for all strains) 8-56 weeks; Embryos E9.5-E17.5.
Wild animals	No wild animals were used in this research.
Field-collected samples	No field-collected samples were used in this research.
Ethics oversight	All animal experiments were compliant with the UK Animals (Scientific Procedures) Act 1986 and approved by the University of Oxford animal welfare review board and the Home Office (project license PB01E1FB3) and following Animals in Research: Reporting in vivo Experiments (ARRIVE) guidelines.

Note that full information on the approval of the study protocol must also be provided in the manuscript.
